# A squalene synthase-like enzyme initiates production of tetraterpenoid hydrocarbons in *Botryococcus braunii* Race L

**DOI:** 10.1038/ncomms11198

**Published:** 2016-04-06

**Authors:** Hem R. Thapa, Mandar T. Naik, Shigeru Okada, Kentaro Takada, István Molnár, Yuquan Xu, Timothy P. Devarenne

**Affiliations:** 1 Department of Biochemistry and Biophysics, Texas A&M University, College Station, Texas 77843, USA; 2 Biomolecular NMR Laboratory, Department of Biochemistry and Biophysics, Texas A&M University, College Station, Texas 77843, USA; 3 Laboratory of Aquatic Natural Products Chemistry, Graduate School of Agricultural and Life Sciences, The University of Tokyo, Yayoi, Bunkyo, Tokyo 113-8657, Japan; 4 Japan Science and Technology Agency-Core Research for Evolutional Science and Technology (CREST), Gobancho, Chiyoda, Tokyo 102-0076, Japan; 5 Natural Products Center, School of Natural Resources and the Environment, The University of Arizona, Tucson, Arizona 85739, USA; 6 Biotechnology Research Institute, The Chinese Academy of Agricultural Sciences, Beijing 100081, China

## Abstract

The green microalga *Botryococcus braunii* is considered a promising biofuel feedstock producer due to its prodigious accumulation of hydrocarbon oils that can be converted into fuels. *B. braunii* Race L produces the C_40_ tetraterpenoid hydrocarbon lycopadiene via an uncharacterized biosynthetic pathway. Structural similarities suggest this pathway follows a biosynthetic mechanism analogous to that of C_30_ squalene. Confirming this hypothesis, the current study identifies C_20_ geranylgeranyl diphosphate (GGPP) as a precursor for lycopaoctaene biosynthesis, the first committed intermediate in the production of lycopadiene. Two *squalene synthase* (*SS*)-like complementary DNAs are identified in race L with one encoding a true SS and the other encoding an enzyme with lycopaoctaene synthase (LOS) activity. Interestingly, LOS uses alternative C_15_ and C_20_ prenyl diphosphate substrates to produce combinatorial hybrid hydrocarbons, but almost exclusively uses GGPP *in vivo*. This discovery highlights how SS enzyme diversification results in the production of specialized tetraterpenoid oils in race L of *B. braunii*.

## Abstract (web summary)

The green microalga Botryococcus braunii is a promising biofuel producer due to its ability to produce large amounts of hydrocarbon oils that can be converted into fuels. Here the authors implicate lycopaoctaene synthase, a squalene synthases-like enzyme, in the first step towards the biosynthesis of the C40 tetraterpenoid hydrocarbon lycopadiene.

## Graphical abstract

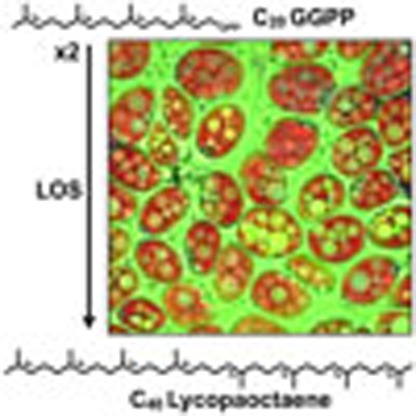

Microalgae are a promising next-generation source of feedstocks for biofuel production with the potential to serve as a practical alternative to petroleum-based transportation fuels^1^. Depending on the microalgal species, the oils produced vary from triacylglycerols to hydrocarbons^2^,^3^. Hydrocarbon-based fuels are preferred over other biofuels, as they are highly compatible with existing petroleum infrastructures and possess superior fuel properties^4^,^5^. The colony-forming green microalga *B. braunii* is an exciting candidate for biofuel feedstock production, as it produces up to 61% of its dry weight as liquid hydrocarbon oils^6^. These hydrocarbons are produced inside the cells of the colony, seen as intracellular oil bodies and secreted into the colony extracellular matrix where the majority of the hydrocarbons are stored^6^ (Fig. 1a). Most importantly, catalytic hydrocracking of hydrocarbons from this alga results in petroleum-equivalent fuels of gasoline, kerosene and diesel^7^. Intriguingly, geologic evidence also shows a direct contribution of this alga to the formation of currently used fossil fuel deposits around the globe^8^,^9^,^10^,^11^,^12^. Despite the aforementioned advantages of *B. braunii*, its use for biofuel feedstock production is hindered by a slow growth rate and the lack of transformation systems to achieve targeted genetic modification^3^. Thus, the identification of *B. braunii* hydrocarbon biosynthetic pathways and associated genes/enzymes can provide options for metabolically engineering these pathways into heterologous hosts with better growth characteristics and the ability to be genetically manipulated. This would then allow the development of improved versions of hydrocarbon biosynthetic enzymes, to direct production towards the most commercially desirable products^13^.

There are three different races of *B. braunii* based on the hydrocarbons synthesized. Race A produces fatty acid-derived C_23_–C_33_ alkadienes and alkatrienes. Races B and L produce isoprenoid-derived hydrocarbons: methylsqualenes and C_30_–C_37_ botryococcene triterpenoids in race B and the C_40_ tetraterpenoid lycopadiene, the focus of this study, in race L^6^. Green algae have been shown to possess only the plastid-localized methyl erythritol phosphate pathway to supply isoprenoid precursors for terpene production^14^,^15^, and thus lycopadiene is predicted to be generated from C_5_ precursors via this route. However, the exact mechanism of lycopadiene biosynthesis from C_20_ prenyl diphosphate intermediates has been a mystery and two possible biosynthetic routes have been suggested (Fig. 1b)^16^. The first entails C_20_ geranylgeranyl diphosphate (GGPP) reduction by GGPP reductase to produce C_20_ phytyl diphosphate (PPP; Fig. 1b). Two molecules of PPP would then undergo head-to-head condensation (1-1′ linkage) to produce lycopadiene (Fig. 1b). The second possibility is the head-to-head condensation of two GGPP molecules to produce lycopaoctaene, followed by stepwise enzymatic reduction to produce lycopadiene (Fig. 1b).

Using either proposed route, the condensation of PPP or GGPP is predicted to proceed in a reaction mechanism similar to that carried out by the enzyme squalene synthase (SS), which forms C_30_ squalene, a precursor required for sterol biosynthesis in eukaryotes^17^ and hopanoids in some prokaryotes^18^. SS enzymes catalyse a two-step reaction. First, the condensation of two C_15_ farnesyl diphosphate (FPP) molecules yields the cyclopropyl intermediate presqualene diphosphate (PSPP; Fig. 1c). Second, PSPP undergoes NADPH-dependent reductive rearrangement to form squalene with a 1-1′ linkage between the two FPP molecules (Fig. 1c)^19^. Herein we report the elucidation of the first committed step in the lycopadiene hydrocarbon biosynthetic pathway in *B. braunii* Race L, which is catalysed by a new SS-like (SSL) enzyme.

## Results

### L race hydrocarbon content and related enzyme activity

Previous studies reported *trans,trans*-lycopadiene as the predominant hydrocarbon (98% of total hydrocarbons) produced by race L, with a small amount of lycopatriene also documented^16^,^20^,^21^. However, the lycopatriene structure was not determined. Thus, we first set out to ascertain and refine the hydrocarbon profile of *B. braunii* Race L, to provide a baseline for our biosynthetic models. Analysis of purified hydrocarbons by gas chromatography–mass spectrometry (GC–MS) showed that lycopadiene accounts for 95% of the total hydrocarbon pool (Fig. 2a (I)), with six other minor compounds constituting the remaining 5%: lycopatriene, lycopatetraene, lycopapentaene, lycopapentaene isomer, lycopahexaene and a C_35_H_64_ molecule (Fig. 2a (II–VII) and Supplementary Figs 1–4). Ozonolysis experiments suggested lycopatriene and lycopapentaene share an identical reduced C_20_ moiety with lycopadiene (Supplementary Figs 5–7). Nuclear magnetic resonance (NMR) spectroscopy was used to confirm identity and structure, including double bond positions, of each molecule (Fig. 2a, Supplementary Table 1 and Supplementary Figs 8–15). This is the first report of lycopatetraene, lycopapentaene and lycopahexaene hydrocarbons from *B. braunii* race L. The lack of detectable amounts of lycopaoctaene and lycopaheptaene in the total hydrocarbon fraction could be due to rapid conversion of these metabolites into more highly saturated homologues. Interestingly, the unique C_35_H_64_ isoprenoid hydrocarbon detected contains seven isoprene units and thus is assigned to the recently named C_35_ terpene class ‘sesquarterpenes'^22^.

**Figure 1:**
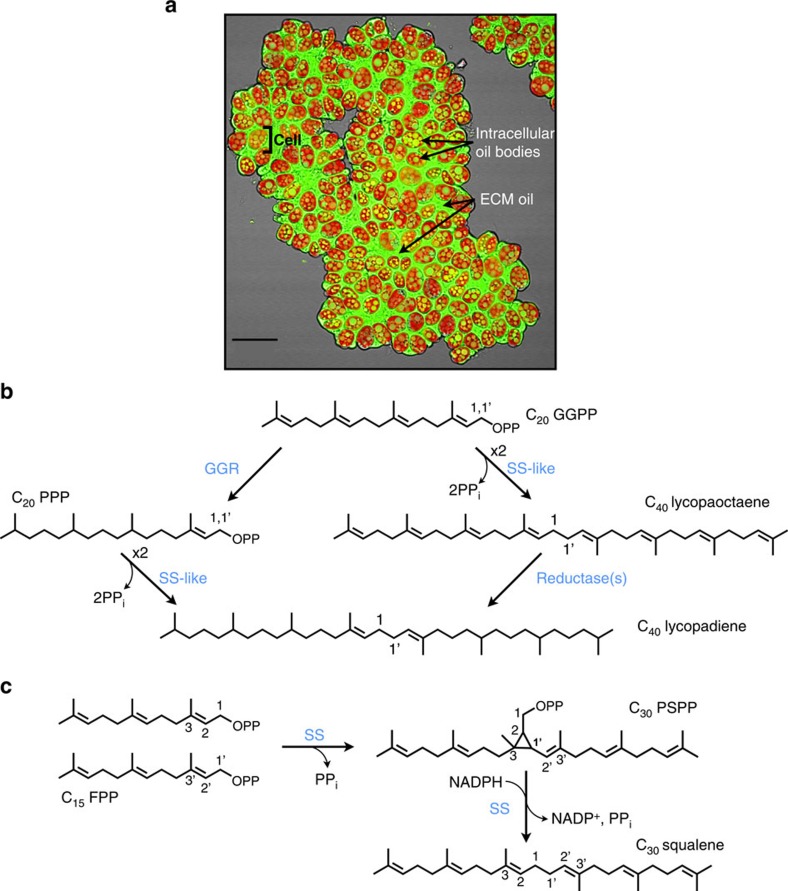
Background information on lycopadiene biosynthesis in *B. braunii* race L. (**a**) Confocal microscopy image of an L race colony of *B. braunii*. Shown are hydrocarbon oils stained using the neutral lipid-binding stain Nile red (false coloured green) and chlorophyll autofluorescence (red). Indicated are intracellular hydrocarbon oil bodies, hydrocarbon oil in the extracellular matrix (ECM) and an individual cell as defined by chlorophyll autofluorescence from the single chloroplast in each cell. Image is an overlay of Nile red signal, chlorophyll autofluorescence signal and a bright-field image. Scale bar, 20μm. (**b**) Two possible pathways for lycopadiene biosynthesis. Possibility 1: GGPP reduction to PPP followed by condensation of two PPP molecules, to directly produce lycopadiene. Possibility 2: condensation of two molecules of GGPP to form lycopaoctaene, which would then be reduced to lycopadiene. (**c**) Two-step reaction catalysed by single SS enzyme: two molecules of FPP are condensed to squalene through the PSPP intermediate. In **b** and **c**, enzyme names are coloured blue.

**Figure 2:**
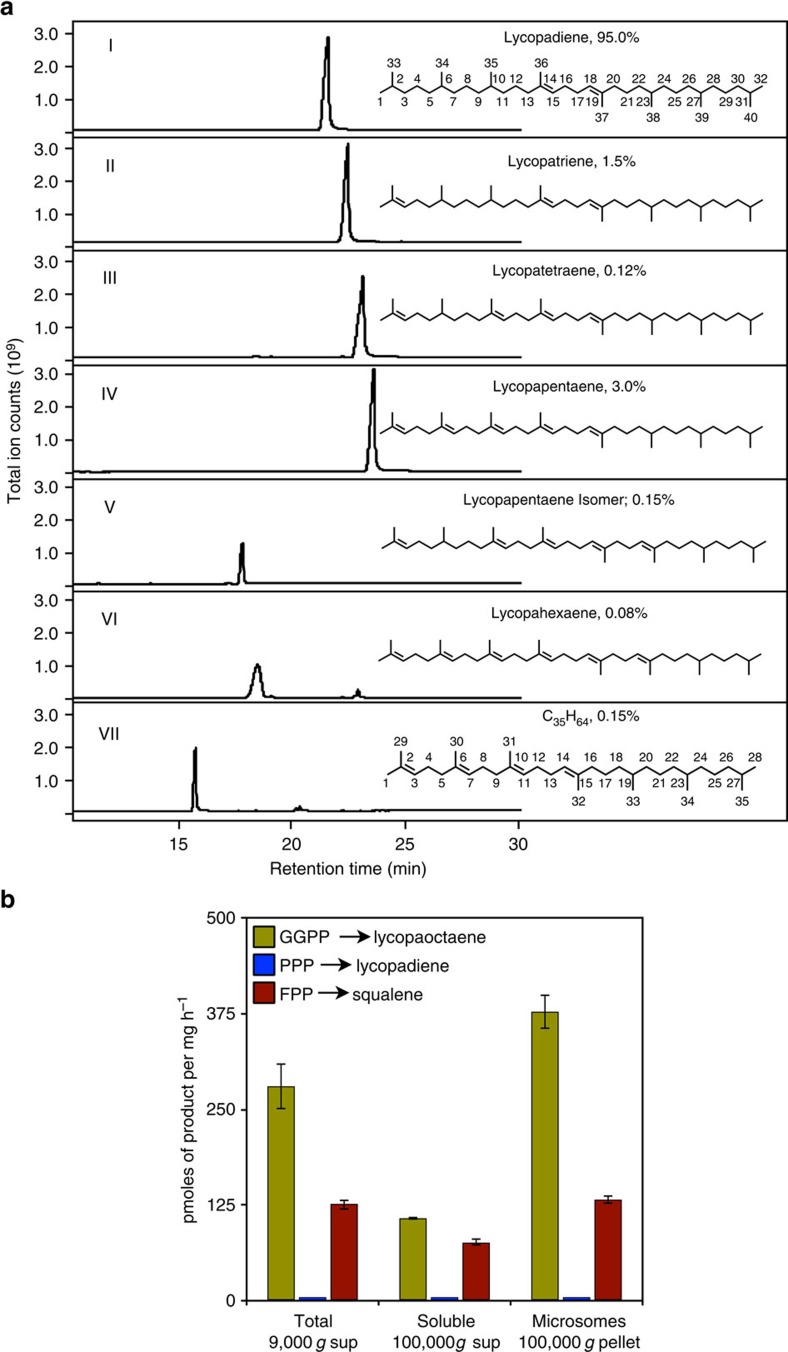
Hydrocarbon composition and hydrocarbon-related enzyme activities in *B. braunii* race L. (**a**) Representative GC–MS profiles of purified hydrocarbons and their corresponding names and structures (I–VII). Structures of hydrocarbons were determined by ^1^H- and ^13^C-NMR. Lycopadiene carbon numbering (I) is used for numbering all C_40_ hydrocarbons of race L. Percentage next to the name of each hydrocarbon corresponds to its average amount present in total hydrocarbon pool from three independent culture cycles (*n*=3). (**b**) Radioactive enzyme assays were conducted using different protein fractions of race L cell lysates to test the two possible lycopadiene biosynthetic pathways. Enzyme activities were calculated based on the incorporation of ^3^H-GGPP into lycopaoctaene, ^3^H-PPP into lycopadiene and ^3^H-FPP into squalene. SS enzyme activity is used as a positive control. Values shown are the mean±s.e. obtained from three independent experiments (*n*=3).

To gain more insight into the mechanism of lycopadiene biosynthesis and assess the validity of the two possible lycopadiene biosynthetic routes (Fig. 1b), a cell-free *in vitro* enzyme assay was developed using total, soluble and microsomal protein fractions with ^3^H-labelled PPP and GGPP substrates for potential incorporation into lycopadiene and lycopaoctaene, respectively. Squalene production by SS enzyme activity with ^3^H-labelled FPP as substrate was used as a control^23^. Lycopadiene production from PPP was not detected in any protein fraction tested, whereas lycopaoctaene biosynthesis from GGPP was easily observed, mainly in the microsomal fraction (Fig. 2b). This indicates a lycopaoctaene synthase (LOS) activity is localized to a membrane system, possibly the endoplasmic reticulum (ER) as is seen for SS^24^. Tellingly, LOS activity in these assays was detected when using the same cofactor and divalent metal cation as those used for SS activity measurement. These results suggest direct lycopadiene biosynthesis from PPP is not a significant contributor to C_40_ hydrocarbon production in race L, whereas conversion of GGPP into lycopaoctaene occurs readily, as proposed in the second hypothetical route (Fig. 1b). Correspondingly, LOS enzyme activity directly correlated with lycopadiene accumulation over the growth cycle, suggesting this activity is related to hydrocarbon biosynthesis (Supplementary Fig. 16). Detection of LOS activity from an algal homogenate in an assay similar to that of SS supports the notion that an LOS enzyme may be similar to a typical SS enzyme.

### Identification of an LOS enzyme

To identify the gene(s) responsible for lycopaoctaene biosynthesis, we generated and computationally screened an L race transcriptomic database for *SSL* sequences. Two *SSL* cDNAs were identified and named based on the function of their encoded proteins as detailed below: *SS* from race L (*LSS*) and *LOS*. Both the LSS and LOS proteins contain all five conserved activity domains, the transmembrane domain and the NADPH-binding residues found in typical SS enzymes (Supplementary Figs 17 and 18)^17^. Comparison of the encoded amino acid sequences showed 57.5 and 49.2% identity to SS from race B (BSS)^25^ for LSS and LOS, respectively (Supplementary Fig. 17). In addition, comparison of LOS with other SS and SSL enzymes showed 52.5% identity to LSS, 47.3% identity to the *Solanum lycopersicum* (tomato) SS (*Sl*SS), 44.6, 61.7 and 44.1% identity to the SSL-1, -2 and -3 from the B race of *B. braunii*, and 33.9% identity to the human SS (HSS; Supplementary Fig. 18). Several studies^17^,^26^,^27^,^28^, including the recent determination of the crystal structure of HSS^29^, have elucidated the catalytic mechanism of the SS reaction. These studies have shown the FLAP domain, domains I–V and the JK loop of HSS form the SS active site^29^. Furthermore, several HSS residues (Ser51, Sr53, Tyr171 and Gln212), two DXXED motifs in domains II and IV, and the NADPH-binding residues were determined to be key for catalytic activity (see Supplementary Fig. 18)^29^. All of these catalytic residues and motifs are also conserved in both the LSS and LOS enzymes (Supplementary Fig. 18), making it difficult at this time to determine which domains/residues are specific for LOS activity in a comparison of LSS and LOS.

**Figure 3:**
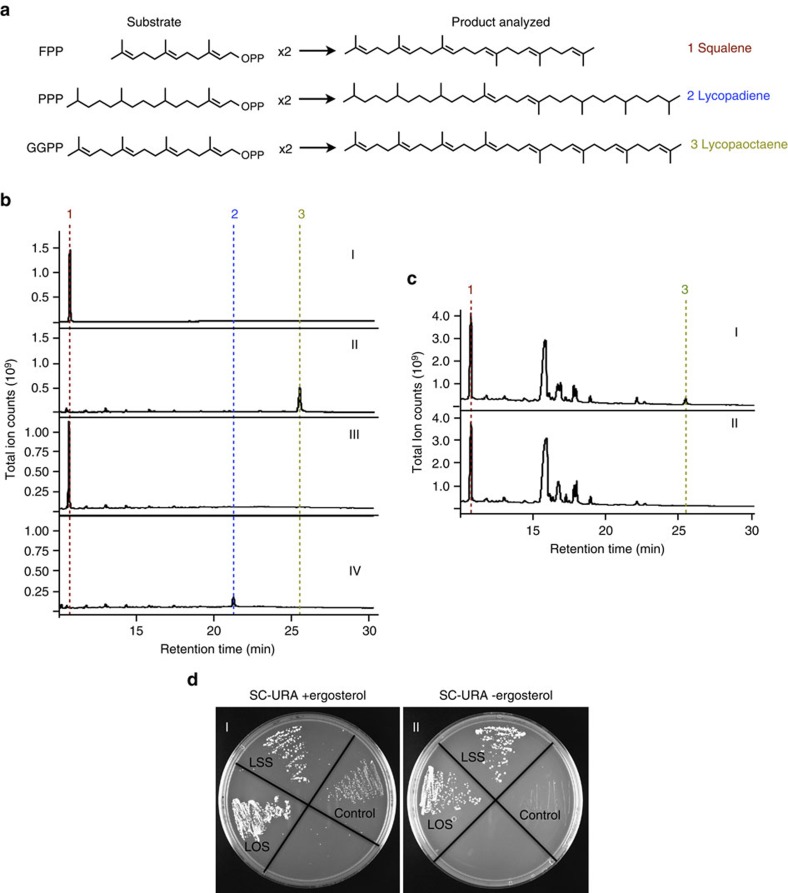
Functional characterization of SSL enzymes from race L *in vitro* and *in vivo*. (**a**) Diagram of reactions showing substrates and products for the analysis in **b** and **c**. (**b**) GC–MS profiles of LSS and LOS *in vitro* reaction products: I, LSS products using FPP as substrate to produce squalene (1); II, LOS products using GGPP as substrate to produce lycopaoctaene (3); III, LOS products using FPP as substrate to produce squalene (1); and IV, LOS products using PPP as substrate to produce lycopadiene (2). (**c**) GC–MS profiles of LOS yeast *in vivo* reaction products: I, *n*-hexane extractable metabolites of yeast expressing *At*GGPPS11 and LOS, to make lycopaoctaene (3); or II, yeast expressing *At*GGPPS11 alone. (**d**) Expression of LSS and LOS in the *SS* (*ERG9*) yeast knockout strain: I, in the presence of ergosterol, or II, in the absence of ergosterol, to test restoration of ergosterol prototrophy. The control carries the expression vector without a transgene. Data shown in **b**,**c** and **d** are representatives from three independent experiments (*n*=3).

For initial characterization of the enzymatic activity of LSS and LOS, both proteins were expressed in *Escherichia coli* and the purified recombinant proteins tested for enzyme activity. LSS was shown to yield squalene as its sole product in an *in vitro* assay with FPP as the substrate, as expected (Fig. 3a,b (I)). However, purified recombinant LOS incubated with GGPP in an *in vitro* assay showed lycopaoctaene as the sole reaction product (Fig. 3a,b (II)). As LOS may have arisen from an SS paralogue that evolved to accept GGPP as substrate for lycopaoctaene production, we considered that LOS may have retained the ability to use FPP to produce squalene. Indeed, squalene production was detected when LOS was incubated with FPP in an *in vitro* reaction (Fig. 3a,b (III)). More surprising, LOS incubation with PPP yielded lycopadiene, albeit at levels much lower than lycopaoctaene or squalene (Fig. 3a,b (IV)). Similar *in vitro* incubations of LSS with GGPP or PPP did not result in lycopaoctaene or lycopadiene production (see below). As LOS can use three different substrates, steady-state kinetic experiments were performed with FPP, GGPP and PPP, to analyse LOS substrate specificity. The LOS enzyme shows higher substrate affinity (*K*_m_) for GGPP compared with that of FPP and PPP, and the turnover number (*k*_cat_) and catalytic efficiency (*k*_cat_/*K*_m_) for PPP are an order of magnitude less than for FPP and GGPP (Table 1 and Supplementary Fig. 19).

To further characterize LOS, it was coexpressed in yeast with *Arabidopsis thaliana* GGPP synthase-11 (*At*GGPPS11)^30^, as unlike FPP, yeast GGPP biosynthesis is limited and is considered a bottleneck for GGPP-derived isoprenoid production^31^. Coexpression of LOS and *At*GGPPS11 in yeast resulted in lycopaoctaene production (Fig. 3c (I)), which was undetectable when *At*GGPPS11 was expressed without LOS (Fig. 3c (II)). Furthermore, when expressed in a yeast *SS* knockout strain, LOS restored ergosterol prototrophy, indicating its ability to produce squalene *in vivo* (Fig. 3d (I,II)). LSS was also able to restore ergosterol prototrophy as expected (Fig. 3d (I,II)). Taken together, the results in Fig. 3 suggest LSS is a true SS enzyme, whereas LOS appears to be a promiscuous SSL enzyme with broader substrate chain length and saturation specificity.

Previous studies have reported the production of lycopaoctaene (a.k.a. lycopersene) *in vitro* from protein extracts during studies conducted to decipher the carotenoid biosynthetic pathway^32^,^33^,^34^; however, subsequent studies determined lycopaoctaene could not be an intermediate in carotenoid biosynthesis^35^,^36^, raising the possibility that lycopaoctaene production in these studies was an *in vitro* artefact. A more relevant study reported the ability of purified yeast SS to use GGPP for lycopaoctaene production *in vitro*, at levels much lower than native squalene production^32^. Importantly, we did not detect any lycopaoctaene production when *At*GGPPS11 was expressed alone in wild-type yeast (Fig. 3c (II)), suggesting that yeast SS does not use GGPP *in vivo* under the conditions employed. Similarly, the ability of LOS to catalyse the conversion of PPP to lycopadiene may not have biological significance as we did not detect lycopadiene production using PPP as the substrate *ex vivo* in race L cell lysates (Fig. 2b) and the efficiency of LOS to use PPP as a substrate is quite low (Table 1). Together, these results suggest that the relevant *in vivo* route to lycopadiene production is unlikely to involve the condensation of two PPP molecules by LOS, but rather, as we contend, the condensation of two GGPP molecules to form lycopaoctaene, for eventual conversion to lycopadiene.

### LOS is promiscuous towards prenyl substrates

**Figure 4:**
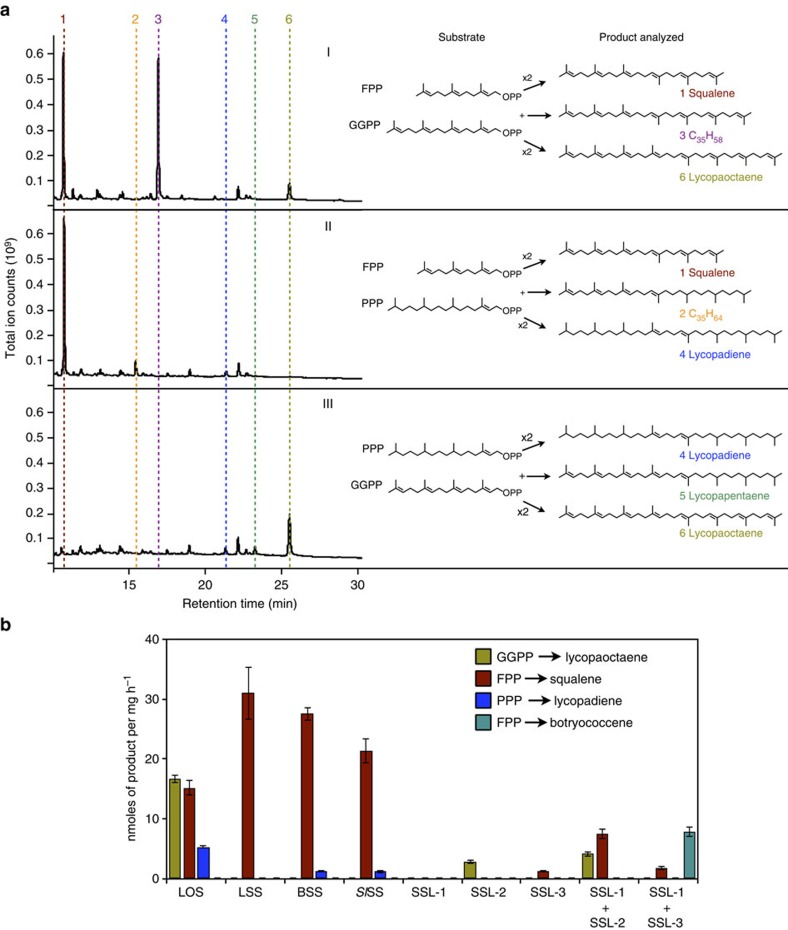
Characterization of substrate use for SS and SSL enzymes. (**a**) Diagram of reactions showing substrates and products, and GC–MS profiles of LOS enzyme assay products with: I, FPP and GGPP substrates to produce squalene (1), C_35_H_58_ (3) and lycopaoctaene (6); II, FPP and PPP substrates to make squalene (1), C_35_H_64_ (2) and lycopadiene (4); and III, GGPP and PPP substrates to produce lycopadiene (4), lycopapentaene (5) and lycopaoctaene (6). Data shown are representatives from three independent experiments (*n*=3). (**b**) Radioactive enzyme assays using ^3^H substrates GGPP, FPP and PPP with selected SS or SSL enzymes. SSL-1 produces PSPP from FPP substrate; however, PSPP was not analysed for in this assay. BSS, race B SS; LOS, lycopaoctaene synthase; LSS, race L SS; *Sl*SS, SS from *S. lycopersicum* (tomato), and SSL-1, SSL-2, SSL-3 are SSL enzymes from the B race of *B. braunii*. Values shown are the mean±s.e. obtained from three independent measurements (*n*=3).

The LOS enzyme was further characterized using combinations of FPP, GGPP and PPP as substrates in *in vitro* reactions. Interestingly, in the presence of FPP and GGPP, LOS produced significant amounts of squalene and a C_35_H_58_ molecule, and lesser amounts of lycopaoctaene (Fig. 4a (I)). The C_35_H_58_ molecule is a chimera produced from head-to-head condensation of one FPP molecule and one GGPP molecule, and its identity was confirmed by GC–MS (Supplementary Fig. 20). Next, when LOS was supplied with FPP and PPP, squalene production predominated with small amounts of C_35_H_64_ and lycopadiene (Fig. 4a (II)). C_35_H_64_, also a chimeric hydrocarbon, is produced as a result of head-to-head condensation of one molecule each of FPP and PPP. Finally, LOS incubation with GGPP and PPP produced lycopadiene and lycopapentaene as minor products and lycopaoctaene as the major product (Fig. 4a (III)).

To the best of our knowledge, this is the first report where a wild-type, non-mutated eukaryotic SS or SSL enzyme has been shown to be able to use three naturally occurring prenyl diphosphate substrates to yield hydrocarbon products in all possible combinations. To support this notion, several SS and SSL enzymes were tested for their ability to use FPP, GGPP and PPP as substrates (Fig. 4b). The enzymes tested included LOS and three typical SS enzymes: LSS, BSS and *Sl*SS. In addition, enzymes SSL-1, SSL-2 and SSL-3 from race B were chosen because of their SSL activities. SSL-1 uses FPP to produce PSPP, which is then converted by SSL-2 to squalene, or by SSL-3 to C_30_-botryococcene as the major product and squalene as a minor product^37^. As shown in Fig. 4b, the typical SS enzymes display SS activity as their main catalytic function, with BSS and *Sl*SS also using PPP to produce minute amounts of lycopadiene. In contrast, the B race SSL enzymes have limited substrate flexibility, allowing SSL-2 and SSL-1 plus SSL-2 to use GGPP to generate minor amounts of lycopaoctaene (Fig. 4b). However, LOS is the only enzyme tested that is able to use all three substrates and to produce significant amounts of lycopaoctaene, squalene and lycopadiene (Fig. 4b).

### The LOS reaction uses a cyclopropyl intermediate

**Figure 5:**
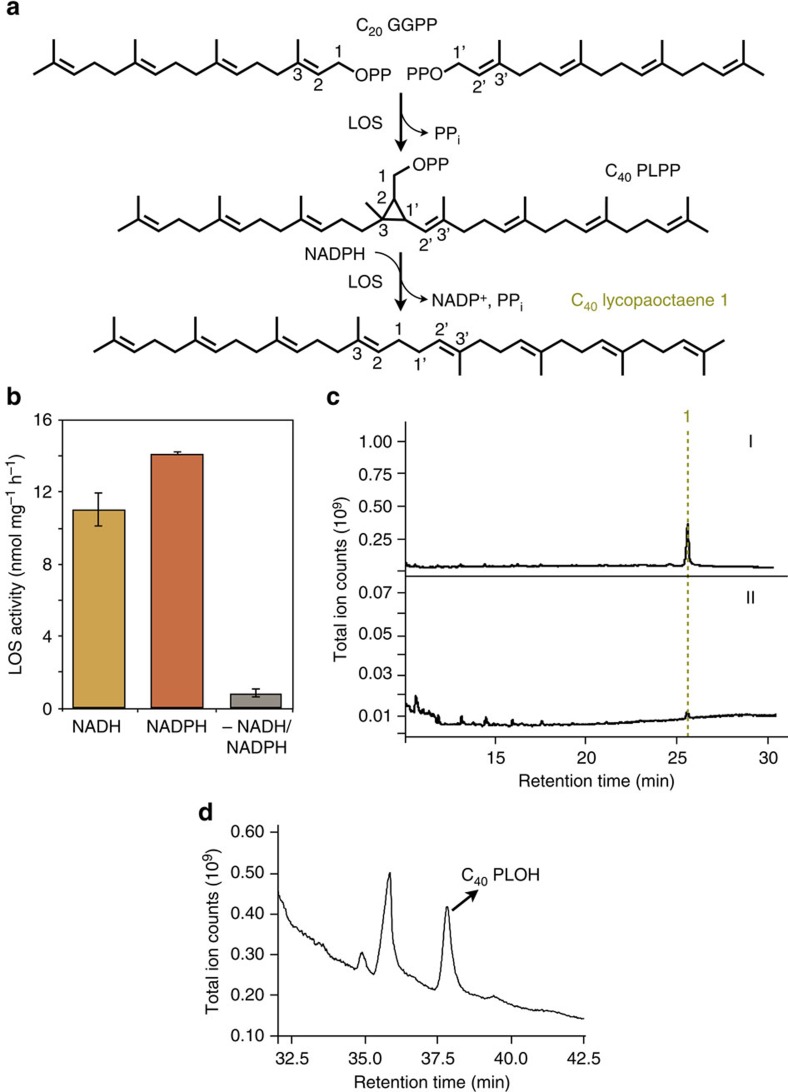
Characterization of the LOS reaction intermediate. (**a**) Proposed LOS reaction mechanism to produce the PLPP intermediate and lycopaoctaene final product. (**b**) Radioactive LOS enzyme assays conducted with ^3^H-GGPP substrate with or without NADH or NADPH reducing agents. Values shown are the mean±s.e. obtained from three independent measurements (*n*=3). (**c**) GC–MS profiles of LOS enzyme assay with GGPP as substrate to produce lycopaoctaene (1): I, in the absence, or II, in the presence of 20 nM squalestatin. (**d**) GC–MS profile of products from LOS enzyme assay with GGPP substrate in the absence of NADPH to produce PLPP, which was hydrolysed by acid phosphatase to yield PLOH for GC–MS analysis. Data shown in **c** and **d** are representatives from three different experiments (*n*=3).

We next conducted enzyme assays to determine whether the LOS reaction mechanism with GGPP as substrate is similar to that of SS, that is, uses a PSPP-like cyclopropyl diphosphate intermediate, which is termed prelycopaoctaene diphosphate (PLPP; Fig. 5a). First, assays were conducted with or without a dinucleotide reducing agent, which would be required to convert PLPP to lycopaoctaene (Fig. 5a). LOS successfully used both NADH and NADPH as reducing agents for lycopaoctaene production, with preference for NADPH (Fig. 5b). In the absence of a dinucleotide reducing agent, LOS activity was lost (Fig. 5b), suggesting the presence of the PLPP reaction intermediate. This result is consistent with previous reports where yeast SS used GGPP *in vitro* to produce lycopaoctaene; however, without NADH or NADPH only the reaction intermediate PLPP accumulated^32^. Next, we showed that LOS is strongly inhibited by squalestatin (Fig. 5c), a potent SS inhibitor that mimics PSPP binding^23^,^38^,^39^,^40^. Finally, we identified PLPP as a reaction intermediate by conducting a GGPP-based LOS assay in the absence of NADPH, followed by acid phosphatase treatment to convert PLPP to prelycopaoctaene alcohol (PLOH). Analysis by GC–MS showed the presence of PLOH (Fig. 5d) with a fragmentation pattern consistent with previous reports of PLOH (Supplementary Fig. 21)^32^. Taken together, these results are consistent with a two-step LOS reaction to produce lycopaoctaene. By analogy to SS, LOS catalyses the condensation of two GGPP units in the first half reaction to form the cyclopropylcarbinyl diphosphate intermediate PLPP, with concomitant release of one molecule of inorganic pyrophosphate (Fig. 5a). In the second half reaction, the PLPP cyclopropyl ring is cleaved and rearranged to form a 1-1′ linkage and further reduction by NADPH forms lycopaoctaene (Fig. 5a).

## Discussion

**Figure 6:**
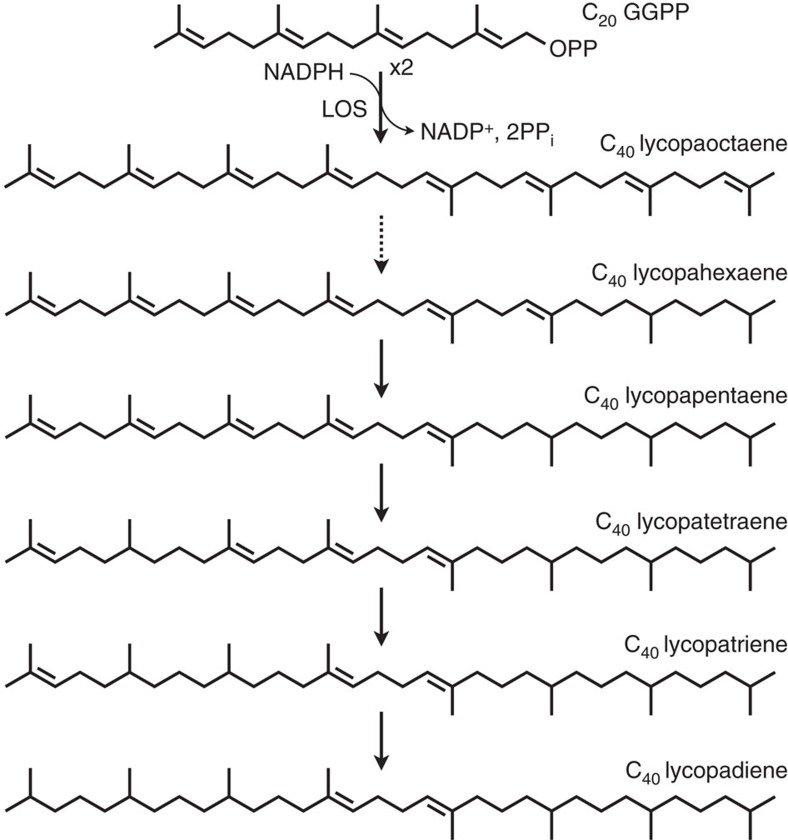
Proposed pathway for lycopadiene biosynthesis. LOS uses GGPP in a two-step reaction to produce lycopaoctaene, which is further reduced by a yet unknown enzyme(s) to produce lycopahexaene, lycopapentaene, lycopatetraene, lycopatriene and finally lycopadiene.

Our studies identified the first committed step in lycopadiene biosynthesis in race L of *B. braunii* and describe a new SSL enzyme, LOS, which carries out this reaction. Importantly, this enzyme may be used to engineer the effective production of hydrocarbon biofuel feedstocks in other photosynthetic or heterotrophic organisms in the future. This novel enzyme produces the tetraterpenoid lycopaoctaene from GGPP in a reaction analogous to that of SS. Lycopaoctaene would then undergo sequential reduction by a yet-to-be identified reductase enzyme(s) to form lycopahexaene, lycopapentaene, lycopatetraene, lycopatriene and finally lycopadiene as the major hydrocarbon product of the pathway (Fig. 6). Furthermore, we show that the wild-type LOS enzyme displays remarkable substrate flexibility and can use at least three different, naturally occurring C_15_ and C_20_ prenyl diphosphate substrates *in vivo* and *in vitro* for the combinatorial biosynthesis of hydrocarbons belonging to three terpene classes: triterpenes, sesquarterpenes and tetraterpenes.

When used under artificial reaction conditions, other SSL enzymes have been described to show relaxed substrate flexibility and to yield products of varying lengths. One such enzyme is CrtM, a dehydrosqualene synthase from *Staphylococcus aureus* that is involved in C_30_ carotenoid biosynthesis^41^,^42^,^43^. In *S. aureus* and also when overexpressed in *E. coli*, CrtM uses two FPP molecules to produce dehydrosqualene, a C_30_ squalene-like molecule with a *cis* double bond at the 1-1′ linkage of the two FPP molecules^44^. Expression of a GGPP synthase in *E. coli* also expressing CrtM led to the depletion of FPP and the overproduction of GGPP^41^. Under these conditions, CrtM was found to generate its native C_30_ product, a C_35_ homologue and C_40_ phytoene, indicating the ability of CrtM to accept GGPP as a substrate when the natural FPP substrate is limiting^41^. CrtM was further engineered through mutations to accept C_25_ farnesylgeranyl diphosphate, yielding C_40_, C_45_ and C_50_ homologues of dehydrosqualene in the presence of FPP, farnesylgeranyl diphosphate and GGPP^42^,^43^. In another study, SSs from yeast, pig and rat have been shown to accept non-natural derivatives of FPP, to generate several unnatural terpene products *in vitro*^45^,^46^. In addition, yeast SS has been shown to yield alternative products *in vitro* from FPP as a substrate under various non-physiological reaction conditions, such as in the absence of NADPH with extended incubation times or in the presence of an unreactive NADPH analogue^47^,^48^,^49^. Irrespective of these examples, the promiscuity of LOS is still remarkable, considering that the wild-type LOS enzyme is able to accept three naturally occurring prenyl diphosphate substrates and produce the chimeric hydrocarbons described here without the need for mutations or altered, non-physiological reaction conditions. Such remarkable intrinsic substrate promiscuity has not been documented for any other eukaryotic SS or SSL enzyme up until now.

Ours and other studies^37^,^50^ raise the question about how SSL enzymes arise. LSS and LOS from *B. braunii* share >50% sequence identity and both contain the important catalytic residues and conserved domains of SS enzymes (Supplementary Fig. 18), but they still have different substrate preferences and product formation. Thus, we hypothesize that an ancient *SS* gene may have undergone gene duplication in race L. The paralogue that gave rise to LSS maintained the ability to produce squalene as its sole product to support sterol biosynthesis for primary metabolism. However, the paralogue that yielded LOS developed the use of alternative substrates for hydrocarbon oil production, while also preserving SS activity. A similar scenario has been suggested as a possible mechanism for generating new secondary metabolites that may provide a fitness benefit to the host organism^42^,^51^. For *B. braunii*, it has been proposed that hydrocarbon oils and their derivatives increase the buoyancy of colonies for increased exposure to sunlight^50^,^52^,^53^. Similar gene diversifications have also been proposed in the B race of *B. braunii* for botryococcene production, and in some bacteria for squalene generation. For botryococcene production, *SS* duplications gave rise to the *SSL-1* and *SSL-3* genes/enzymes responsible for botryococcene production, while retaining a residual ability to produce squalene^37^. For bacteria, a three-enzyme system for squalene production arose from successive gene duplication events^50^.

LOS produces a variety of terpenes *in vitro* when offered a mixture of substrates and may likely also do so *in vivo*. In the *in vitro* mixed substrate assays with LOS (Fig. 4a), a C_35_H_64_ molecule was observed from FPP and PPP condensation. This product has the exact same GC retention time and mass spectrum as the C_35_H_64_ hydrocarbon detected in the total hydrocarbon pool (Fig. 2a (VII) and Fig. 4a (II)). This suggests that LOS is also promiscuous in *B. braunii* cells, but at best has limited access to the FPP and PPP pools, as the C_35_H_64_ product only comprises 0.15% of the total hydrocarbons (Fig. 2a (VII)). The FPP–GGPP condensation product C_35_H_58_ seen in these *in vitro* assays (Fig. 4a (I)) is not detected in the total hydrocarbon pool, possibly because this molecule is reduced to C_35_H_64_ *in vivo*. It should be noted that two previous studies described several C_35_ squalene-like isoprenoids from *Methanococcus jannaschii* and *Thermococcus barophilus*, including the C_35_H_58_ and C_35_H_64_ molecules identified here^54^,^55^, although the enzymatic basis for the biosynthesis of these products remains uncharacterized. In addition, the ability of LOS to produce lycopapentaene as an observed *in vitro* condensation product of GGPP and PPP (Fig. 4a (III)) may indicate the existence of an alternative *in vivo* biosynthetic route to this molecule and may be partially responsible for the relative prevalence of lycopapentaene (3% of total hydrocarbons; Fig. 2a (IV)) among the minor hydrocarbons of race L. This would suggest that LOS can also use PPP *in vivo*. However, LOS does not appear to use PPP as a single substrate for lycopadiene production *in vivo*, as we were not able to detect lycopadiene synthesis activity in cell fractions using PPP as substrate (Fig. 2b).

The promiscuous LOS described in this work is nevertheless used by *B. braunii* Race L to produce lycopaoctaene preferentially over squalene, as shown by the large amounts of hydrocarbon oils produced by this alga and the absence of detectable amounts of squalene or non-sterol squalene derivatives in the total hydrocarbon pool. In contrast, accumulation of the non-sterol squalene derivatives methylsqualenes in race B are thought to arise from squalene produced by the additional enzyme SSL-2 (ref. 37). Thus, detection of non-sterol squalene derivatives in race L would be expected if LOS was producing squalene *in vivo*.

We hypothesize that the observed preference of LOS for C_40_ lycopaoctaene biosynthesis over C_30_ squalene production *in vivo* may result from an increased flux from GGPP over FPP due to metabolic channelling, to form a biosynthetic metabolon specific for lycopadiene production. Formation of such metabolons is common in plants and is used for channelling substrates and intermediates required for the biosynthesis of different classes of plant natural products, including isoprenoids^56^,^57^,^58^. When expressed in yeast, LOS may produce squalene presumably due to a lack of this proposed metabolon, thus giving LOS access to the FPP pool. We therefore conclude that LOS is responsible for synthesizing lycopaoctaene as the first committed step towards lycopadiene hydrocarbon biosynthesis in race L of *B. braunii*.

## Methods

### Reagents

[1-^3^H]-FPP (specific activity, 18.2 Ci mmol^−1^) was purchased from PerkinElmer. [1-^3^H]-GGPP (specific activity, 20.0 Ci mmol^−1^), [1-^3^H]-PPP (specific activity, 20.0 Ci mmol^−1^) and non-radiolabelled PPP were purchased from American Radiolabeled Chemicals. When required, specific activities of radiolabelled chemicals were adjusted with non-labelled chemicals. Solvents and chemicals were purchased from VWR. All other reagents were purchased from Sigma unless otherwise noted.

### Culturing of *B. braunii*

*B. braunii*, race L, Songkla Nakarin strain^16^ was obtained from Algobank-Caen Microalgal Culture Collection, University of Caen Basse-Normandie, France, and grown in a modified Chu 13 medium, pH 7.5 (ref. 59) at 22 °C under continuous aeration of filter-sterilized air enriched with 2.5% CO_2_. The concentrations of chemicals in modified Chu 13 medium were as follows: KNO_3_ (0.4 g l^−1^), MgSO_4_·7H_2_O (0.1 g l^−1^), K_2_HPO_4_ (0.052 g l^−1^), CaCl_2_·2H_2_O (0.054 g l^−1^), FeNa EDTA (0.01 g l^−1^), H_3_BO_4_ (2.86 mg l^−1^), MnSO_4_·H_2_O (1.54 mg l^−1^), ZnSO_4_·7H_2_O (0.22 mg l^−1^), CuSO_4_·5H_2_O (0.08 mg l^−1^), NaMoO_4_·2H_2_O (0.06 mg l^−1^) and CoSO_4_·7H_2_O (0.09 mg l^−1^). The cultures were grown under a 12:12 h light:dark cycle with a light intensity of 120 μE m^−2^ s^−1^. Algae cells were subcultured by inoculation of 100 ml of a floating 6-week-old culture to 750 ml of fresh medium.

### Hydrocarbon staining and microscopy

Nile Red (Sigma) staining of L race hydrocarbons was done by treating 500 μl of *B. braunii* L race colonies in medium at early phase (5 day) density with 2.5 μl of a stock solution of Nile Red dissolved in acetone (0.15 mg ml^−1^) for a final concentration of Nile red of 0.375 g ml^−1^ and acetone of 0.25%. Samples were kept in the dark and incubated at room temperature for 15 min before imaging. Confocal microscopy images were obtained using an Olympus IX83-FV1200 inverted confocal microscope with spectral detection unit using an Olympus UPLSAPO × 60 oil-immersion objective (numerical aperture=1.35). The confocal aperture diameter was set to 1 Airy disk unit and *Z*-series images were acquired with voxel dimensions of 101 × 101 × 490 (nm; *x*-*y*-*z*, respectively). Nile Red and chlorophyll were excited using a 488-nm laser and detected using 540–590 nm and 655–755 nm barrier filter settings, respectively. Bright-field images were acquired via the transmitted detector using the 488-nm laser. Maximum intensity projections and brightness/contrast adjustments were completed using the Olympus FV-ASW software version 4.

### Hydrocarbon purification

Hydrocarbons were purified as described previously^60^,^61^ and as follows. Algal cells were harvested by vacuum filtration using a 10-μm nylon-mesh filter, freeze-dried, extracted twice with *n*-hexane for 2 h, to recover extracellular hydrocarbons, and then twice with CHCl_3_:MeOH (2:1) for 12 h stirring at room temperature, to recover intracellular hydrocarbons. Both extracts were concentrated separately using a rotary evaporator and resuspended in a small amount of *n*-hexane before running through separate gravity-fed silica gel columns with *n*-hexane as solvent. The *n*-hexane eluent before the pigment front was collected as the hydrocarbon fraction. Both extracellular and intracellular hydrocarbon fractions were combined and evaporated to dryness, to recover the total hydrocarbon pool of the algae. Individual hydrocarbon molecules were purified by injecting the total hydrocarbon sample onto an HPLC Develosil 60 silica column (20 mm × 250 mm) using *n*-hexane as a mobile phase at a flow rate of 6 ml min^−1^ and detection at 210 nm. An aliquot of the purified hydrocarbons were analysed by GC–MS for purity analysis.

### GC–MS conditions

Enzyme assay products, purified hydrocarbons and yeast extracts were analysed by GC–MS (Bruker 436-GC-SCION SQ Premium) using a 5% Phenyl BR-5 ms capillary column (30 m × 0.25 mm, film thickness: 0.25 μm) in electron ionization (70 eV) mode. Initial oven temperature was 220 °C, held for 1 min, then increased to 280 °C at the rate of 5 °C min^−1^ and then ramped to 300 °C at a rate of 2 °C min^−1^ and held for 20 min. Helium was used as a carrier gas at a flow rate of 2.58 ml min^−1^. Temperatures of injection port, interface and ion source were 280 °C, 250 °C and 200 °C, respectively.

### Hydrocarbon NMR analysis

One-dimensional NMR spectra of race L hydrocarbons molecules suffer from severe spectral overlap and the data interpretation is further complicated by degenerate chemical shifts as a result of symmetry planes in the lycopadiene structure. Consequently, hetero-nuclear two-dimensional (2D) experiments were used for unambiguous structural characterization of each entity. NMR experiments were performed at 25 °C on purified samples dissolved in deuterated chloroform using a 500 MHz Bruker Avance III HD spectrometer equipped with an inverse detection TXI probe. We have relied on 2D heteronuclear experiments acquired at natural abundance of ^13^C nuclei, to deduce structures of these molecules. Multiplicity-edited 2D heteronuclear single quantum coherence spectrum was acquired for each compound and the resonances were assigned using a combination of 2D heteronuclear 2 bond correlation, 2D heteronuclear multiple bond correlation and 2D heteronuclear single quantum coherence–total correaltion spectroscopy experiments. The ^13^C chemical shifts obtained for lycopadiene match those previously obtained^16^.

### Spectral data for hydrocarbons analysed

Lycopadiene: ^1^H NMR (500 MHz, CDCl_3_): *δ* 0.87, 0.89, 1.08, 1.09, 1.16, 1.2, 1.29, 1.39, 1.4, 1.54, 1.61, 1.96, 2.04, 2.17, 5.15; ^13^C NMR (125 MHz, CDCl_3_): *δ* 15.9, 19.7, 22.7, 24.5, 24.7, 25.4, 28.0, 28.3, 31.2, 32.8, 36.8, 37.4, 39.4, 40.0, 124.0, 135.9; MS (*m/z*): [M^+^] calcd. for C_40_H_78_, 558.61; found, 558.70. Lycopatriene: ^1^H NMR (500 MHz, CDCl_3_): *δ* 0.87, 0.89, 1.08, 1.09, 1.13, 1.16, 1.2, 1.29, 1.29, 1.39, 1.4, 1.54, 1.61, 1.63, 1.71, 1.96, 1.98, 2.04, 2.16, 5.12, 5.15; ^13^C NMR (125 MHz, CDCl_3_): *δ* 15.9, 17.6, 19.7, 22.7, 24.5, 24.7, 24.7, 25.4, 25.6, 25.7, 28.0, 28.3, 30.8, 32.7, 36.8, 37.1, 37.4, 39.4, 40.0, 124.1, 125.1, 131.1, 135.9; MS (*m/z*): [M^+^] calcd. for C_40_H_76_, 556.59; found, 556.70. Lycopatetraene: ^1^H NMR (500 MHz, CDCl_3_): *δ* 0.87, 0.89, 1.08, 1.13, 1.16, 1.2, 1.29, 1.39, 1.4, 1.54, 1.61, 1.63, 1.71, 1.95, 1.96, 1.99, 2.04, 2.16, 5.12, 5.15; ^13^C NMR (125 MHz, CDCl_3_): *δ* 15.9, 17.6, 19.7, 22.7, 24.5, 24.7, 25.4, 25.5, 25.7, 28.0, 28.3, 30.8, 32.7, 36.6, 37.1, 37.4, 39.3, 39.4, 40.0, 124.1, 125.2, 131.1, 135.9; MS (*m/z*): [M^+^] calcd. for C_40_H_74_, 554.58; found, 554.60. Lycopapentaene: ^1^H NMR (500 MHz, CDCl_3_): *δ* 0.87, 0.89, 1.08, 1.16, 1.2, 1.29, 1.39, 1.4, 1.55, 1.62, 1.63, 1.71, 2, 2.04, 2.09, 2.23, 5.15; ^13^C NMR (125 MHz, CDCl_3_): *δ* 16.0, 17.7, 19.8, 22.7, 24.5, 24.7, 25.4, 25.7, 26.7, 28.0, 28.3, 31.0, 32.8, 36.6, 37.4, 39.4, 39.8, 124.2, 131.1, 135.9; MS (*m/z*): [M^+^] calcd. for C_40_H_72_, 552.56; found, 552.70. Lycopapentaene isomer: ^1^H NMR (500 MHz, CDCl_3_): *δ* 0.87, 0.89, 1.08, 1.13, 1.16, 1.2, 1.29, 1.4, 1.55, 1.62, 1.63, 1.71, 1.99, 2, 2.04, 2.09, 5.1, 5.15; ^13^C NMR (125 MHz, CDCl_3_): *δ* 16.0, 17.7, 19.8, 22.7, 24.5, 24.7, 25.5, 25.7, 26.7, 28.0, 28.3, 32.7, 36.6, 37.1, 37.4, 39.4, 39.8, 124.2, 124.8, 131.1, 135.9; MS (*m/z*): [M^+^] calcd. for C_40_H_72_, 552.56; found, 552.90. Lycopahexaene: ^1^H NMR (500 MHz, CDCl_3_): *δ* 0.87, 0.89, 1.08, 1.16, 1.2, 1.29, 1.4, 1.55, 1.61, 1.63, 1.71, 1.96, 1.99, 2.04, 2.06, 5.15; ^13^C NMR (125 MHz, CDCl_3_): *δ* 16.0, 17.7, 19.8, 22.7, 24.5, 24.7, 25.7, 26.8, 28.0, 28.3, 32.8, 36.6, 37.4, 39.4, 39.8, 124.3, 131.1, 135.9; MS (*m/z*): [M^+^] calcd. for C_40_H_70_, 550.55; found, 550.60. Lycopaoctaene: ^1^H NMR (500 MHz, CDCl_3_): *δ* 1.62, 1.63, 1.71, 2, 2.03, 2.04, 5.15; ^13^C NMR (125 MHz, CDCl_3_): *δ* 16.0, 17.7, 25.7, 27.2, 28.3, 39.8, 124.3, 131.1, 135.9; MS (*m/z*): [M^+^] calcd. for C_40_H_66_, 546.52; found, 546.70. C_35_H_64_: ^1^H NMR (500 MHz, CDCl_3_): *δ* 0.83, 0.86, 1.06, 1.13, 1.19, 1.21, 1.24, 1.32, 1.36, 1.52, 1.61, 1.68, 1.98, 2.01, 2.06, 5.13; ^13^C NMR (125 MHz, CDCl_3_): *δ* 16.3, 17.9, 20.0, 22.9, 24.4, 24.8, 25.1, 26.0, 26.3, 27.2, 28.2, 33.0, 37.7, 39.6, 40.0, 124.8, 131.4, 135.1; MS (*m/z*): [M^+^] calcd. for C_35_H_64_, 484.50; found, 484.70.

### Ozonolysis experiments

Owing to the limited amount of minor hydrocarbons in the total L race hydrocarbon pool, ozonolysis experiments were conducted only on those hydrocarbons that could be purified to obtain at least 10 mg: lycopadiene, lycopatriene and lycopapentaene. These hydrocarbons were separately dissolved in dichloromethane and submitted to ozone cleavage for 5 min at −78 °C. Each product from reductive cleavage of the resulting ozonide was directly subjected to GC–MS (electron ionization) analyses without purification. GC–MS analysis was carried out using a GCMS-QP2010 Ultra spectrometer (Shimadzu, Kyoto, Japan) equipped with a capillary column (InertCap 1MS, GL Science; 60 m × 0.25 mm, film thickness: 0.25 μm). The column temperature was programmed as follows: 50 °C for 1 min, raised at 10 °C min^−1^ from 50 to 220 °C, then at 2 °C min^−1^ from 220 to 260 °C and held for 22 min at the final temperature. Helium was used as a carrier gas at a flow rate of 41.2 cm s^−1^. Temperatures of injection port, interface and ion source were 260, 250 and 200 °C, respectively.

### Preparation of algal cell lysate

Algae from the rapid growth phase were harvested, snap frozen with liquid nitrogen and stored at −80 °C for future use. In a typical preparation, 200 mg of frozen algae was added to eight individual tubes containing 0.8 ml extraction buffer (50 mM MOPS pH 6.8, 20 mM MgCl_2_, 5 mM β-mercaptoethanol, 5 mM EGTA and 20% (v/v) glycerol) with 200 μl of stainless steel metal beads (mixture of 0.9–2 mm diameter beads) and then homogenized using a Bullet Blender Storm 24 (Next Advance, Inc., USA) for 10 min at a speed of 10. The algal homogenates were centrifuged at 9,000 *g* for 10 min at 4 °C. The 9,000 *g* supernatants were pooled and then centrifuged at 100,000 *g* for 1 h to obtain the soluble fraction. The pellet was washed with 4 ml extraction buffer and centrifuged again at 100,000 *g*. This process was repeated twice and the pellet was resuspended in 0.5 ml extraction buffer to obtain a microsomal fraction. An aliquot of each protein fraction was used for enzyme assays.

### Enzyme assays

Radioactive enzyme assays were conducted in a 50-μl total reaction volume as described previously^23^,^37^ and as follows. Reactions were initiated by adding 10 μg algal lysate or 1 μg of recombinant enzyme purified from *E. coli* to a reaction buffer containing 50 mM MOPS pH 6.8, 2.5 mM β-mercaptoethanol, 20 mM MgCl_2_, 2 mM NADPH or NADH and 10 μM [1-^3^H]-prenyl-PP substrate (specific activity=0.25 Ci mmol^−1^). Enzyme assays were incubated at 37 °C for 60 min and terminated by adding 60 μl of *n*-hexane, followed by brief vortexing and centrifugation. Thirty microlitres of the organic layer was spotted onto silica gel 60 thin-layer chromatography plates along with authentic standards of squalene (*R*_f_=0.17), C_30_-botryococcene (*R*_f_=0.2), lycopaoctaene (*R*_f_=0.09) and lycopadiene (*R*_f_=0.5), and then developed with *n*-hexane. Hydrocarbon standards were visualized by iodine vapour and spots corresponding to authentic standards were scraped and analysed by liquid scintillation counter, to determine radioactive incorporation into the respective hydrocarbons. SS and botryococcene synthase assays were conducted using ^3^H-FPP, whereas LOS and lycopadiene synthase assays were conducted using ^3^H-GGPP and ^3^H-PPP, respectively. For GC–MS analysis of reaction products, enzyme assays were conducted with non-radiolabelled substrates in glass vials by scaling the total reaction volume and contents to 500 μl. Two separate 500 μl reactions were each extracted twice with 1 ml *n*-hexane, the extracts pooled, dried under a stream of nitrogen gas, resuspended in 100 μl *n*-hexane and a 5-μl aliquot analysed by GC–MS.

### Hydrocarbon accumulation and LOS activity over growth cycle

Several flasks of race L culture were grown over a 39- day culture period. Twenty-five millilitres of culture was collected at indicated times on pre-weighed Whatman GF/C filters by vacuum filtration. The filters with algae were dried in an 80 °C oven and total hydrocarbons were extracted using the protocol described above. Similarly, 500 ml of algae cells were also harvested at indicated times by filtering through a 10-μm nylon mesh and the samples used to determine LOS enzyme activity over the culture period. LOS enzyme assays were conducted using 10 μg of 9,000 *g* supernatant.

### RNA isolation

Total RNA was isolated as previously described^62^ and as follows. For *B. braunii*, liquid nitrogen frozen samples were pulverized using a Tissuelyser II (Qiagen, Valencia, CA). Approximately 200 mg of this frozen pulverized *B. braunii* tissue, or frozen leaf tissues of *A. thaliana* and *S. lycopersicum*, were ground in a mortar and pestle with liquid nitrogen. The samples were then added to 1 ml of TRIzol (Life Technologies, Grand Island, NY) and the total RNA was isolated following the manufacturer's instructions. The RNA from *A. thaliana* and *S. lycopersicum* were used directly for gene cloning (see below). For the *B. braunii* RNA, contaminating polysaccharides were removed before the use for gene cloning as follows. The RNA pellet was resuspended in 0.5 ml 2 M LiCl, incubated for 5 min and centrifuged at 12,000 *g* for 15 min at 4 °C to pellet the total RNA. The polysaccharides remain in the supernatant. This process was repeated until the size of RNA pellet remained constant. The RNA pellet was then dissolved in 0.5 ml 1 × Tris-EDTA (TE), extracted with an equal volume of phenol/chloroform/isoamyl alcohol mix (25:4:1) and centrifuged at 12,000 *g* for 15 min at 4 °C. The aqueous supernatant phase was removed and extracted with 0.5 ml of chloroform followed by centrifugation at 12,000 *g* for 15 min at 4 °C. The RNA was precipitated from the aqueous supernatant by adding 0.1 volume 3 M sodium acetate and 2.5 volumes of 100% ethanol, incubated for 20 min at −20 °C and centrifuged at 12,000 *g* for 15 min at 4 °C. The RNA pellet was washed twice with 0.5 ml 70% ethanol, centrifuged at 12,000 *g* for 15 min at 4 °C, the pellet dried using a speedvac and the RNA resuspended in 50 μl of RNAase-free water.

### *LSS* and *LOS* cloning

RNAseq analysis on the L race of *B. braunii* was carried out as previously reported^62^ and as follows. RNA isolated from days 0, 3, 7, 14, 21 and 28 over the 4-week culture cycle were kept separate, paired-end libraries were prepared from each RNA sample and each sample was sequenced under the Illumina platform. A transcriptome contig library was created using the Trinity software suite. *SSL* sequences were computationally screened using this transcriptomic database with *BSS* as a query. Two *SSL* cDNAs, *LSS* and *LOS*, were identified. Total RNA was extracted from a day-3 race L culture and first-strand cDNA was prepared using the SuperScript III first-strand synthesis kit (Invitrogen). Primers specific to each cDNA based on the transcriptome sequence were used to amplify the PCR product from first-strand cDNA using GoTaq DNA polymerase mix (Promega) followed by cloning into the pGEM-T vector (Promega). Gene-specific primers were as follows: For *LSS*, forward primer 5′- ATGGGGAAGCTACAGGAGGTTTTGAAGC -3′ and reverse primer 5′- TCAGGCAAGGCCGCCGCGAAG -3′; for *LOS*, forward primer 5′- ATGAAGTACACAGATTTCCTTGCGC -3′ and reverse primer 5′- TCACACAGTCTTGAGGGCGAG -3′.

### *AtGGPPS11* and *SlSS* cloning

The cDNA sequences for *AtGGPPS11* (*At*4g36810)^30^ and *SlSS* were obtained from the NCBI nucleotide database. Total RNA isolated from the leaves of *A. thaliana* and *S. lycopersicum* were used for first-strand cDNA synthesis and PCR products corresponding to each cDNA were amplified using gene-specific primers followed by cloning into the pGEM-T vector. Gene-specific primers used for PCR amplification were as follows: for *AtGGPPS11*, forward primer 5′- ATGGCTTCAGTGACTCTAGGTTC -3′ and reverse primer 5′- TCAGTTCTGTCTATAGGCAATG -3′; for *SlSS*, forward primer 5′- ATGGGAACATTGAGGGCA -3′ and reverse primer 5′- CTAAGACCGGCTGCCAAAAAGTTG -3′.

### Protein expression and purification

Except for *SSL-1* and *SSL-3*, DNA sequences encoding the predicted transmembrane domain at the carboxy terminus of each SS or SSL protein were deleted and then cloned to pET28a, to encode for amino terminal 6 × His-tagged proteins. DNA templates of *BSS*, *SSL-1*, *SSL-2* and *SSL-3* were received from previous studies^25^,^37^. The pET28a expression constructs were made using appropriate restriction sites; *LOS*^*Δ391–444*^ with NheI and HindIII; *LSS*^*Δ399–462*^, *SlSS*^*Δ387–411*^, *BSS*^*Δ399–461*^, *SSL-1* and *SSL-3* with NdeI and HindIII; and *SSL-2*^*Δ392–465*^ with NdeI and SalI. The expression constructs were transformed into *E. coli* BL21(DE3), grown at 37 °C to OD_600_=0.8 and protein expression for each gene induced by adding 1 mM isopropylthio-β-D-galactoside. The induced cultures were then grown for an additional 6 h at 25 °C.

His-tagged proteins were purified at 4 °C by standard procedures. In a typical purification, pellets from a 100-ml culture were resuspended in 10 ml extraction buffer (50 mM sodium phosphate buffer pH 7.8, 300 mM NaCl, 10 mM imidazole, 1 × general protease inhibitor cocktail (Sigma), 1 mM MgCl_2_ and 1% glycerol (v/v)), and then sonicated four times for 15 s at 70% maximum power with 2 min interval between each sonication. The sonicated samples were centrifuged at 16,000 *g* for 10 min at 4 °C. The supernatants were applied to a gravity-fed column containing Ni-NTA agarose (Qiagen) and the His-tagged proteins were purified according to the manufacturer's recommendations. The wash buffer (50 mM sodium phosphate buffer pH 7.8, 300 mM NaCl, 60 mM imidazole, 1 mM MgCl_2_ and 1% glycerol (v/v)) and the elution buffer (50 mM sodium phosphate buffer pH 7.8, 300 mM NaCl, 400 mM imidazole, 1 mM MgCl_2_ and 1% glycerol (v/v)) were used for protein purification. The eluted fractions were dialysed using storage buffer (300 mM NaCl, 20 mM Tris-HCl pH 7.5, 5 mM dithiothreitol and 2 mM MgCl_2_), concentrated with an Amicon Ultra centrifugal filter (0.5 ml, 30 kDa cutoff; EMD Millipore) to desired protein concentration, an equal amount of 100% glycerol added and stored at −20 °C for 1–2 months without a loss of enzyme activity.

### LOS steady-state kinetic experiments

Michaelis–Menten enzyme kinetics experiments with the LOS enzyme were set up using the protocol described previously^63^ and as follows. Pilot experiments were initially conducted using radioactive enzyme assays, to determine the reaction conditions where reaction velocity is linear (<10% turnover) with respect to enzyme concentration. For steady-state kinetics experiments, enzyme assays (50 μl total reaction volume) were conducted with 100 nM of purified LOS enzyme at the indicated concentration of ^3^H-GGPP, ^3^H-FPP or ^3^H-PPP for a set time interval. Kinetics for the NADPH cofactor were not determined and this factor was held constant at 2 mM in all assays. The reaction velocities were plotted against substrate concentrations to generate Michaelis–Menten curves and the kinetics parameters of LOS enzyme for GGPP, FPP and PPP were determined by analysing the data using GraphPad Prism 6 software.

### Yeast expression

The DNA sequence encoding the 56 N-terminal amino acids for the plastid-targeting signal of *AtGGPPS11* were deleted resulting in *AtGGPPS11*^*Δ57*^, which was cloned into pESC-TRP using BamHI and SalI restriction sites for expression under the inducible GAL1 promoter. For coexpression studies, the DNA sequences encoding the predicted C-terminal transmembrane domain of *LOS* was deleted resulting in *LOS*^*Δ391–444*^, which was cloned into the second multiple cloning site of *AtGGPPS11*^*Δ57*^:pESC-TRP using EcoRI and SpeI restriction sites for expression under the inducible GAL10 promoter. The yeast expression constructs *AtGGPPS11*^*Δ57*^:pESC-TRP and *AtGGPPS11*^*Δ57*^+*LOS*^*Δ391–444*^:pESC-TRP were introduced into yeast strain CKY457 (*MAT***a**, leu2Δ1, *ura3-52*, *trp1Δ63*, *his3Δ200* and *lys2-128∂*) via lithium acetate transformation followed by selection on yeast synthetic dropout medium (SC-TRP). Positive transformants were grown at 30 °C in 150 ml SC-TRP media to mid-log phase, induced with 2% final galactose concentration and grown for an additional 130 h. Yeast cells were harvested, freeze-dried and extracted with *n*-hexane for 2 h by stirring at room temperature. The organic extracts were centrifuged at 1,000 *g*, the supernatant dried using a rotary evaporator, resuspended in 500 μl *n*-hexane and a 5-μl aliquot analysed by GC–MS to evaluate for the production of lycopaoctaene.

### Purification of lycopaoctaene standard

A 3-l culture of yeast strain CKY457 expressing *AtGGPPS11*^*Δ57*^+*LOS*^*Δ391–444*^:pESC-TRP was grown and the metabolites were extracted with *n*-hexane using the protocol described above. The organic extracts were applied to a silica gel gravity-fed column and metabolites eluted sequentially with two different solvent systems, *n*-hexane and 1% diethyl ether in *n*-hexane. The 1% diethyl ether in *n*-hexane eluent fraction containing lycopaoctaene and other metabolites was concentrated and resuspended in a small volume of *n*-hexane before injecting the samples onto a 10 mm × 250 mm Cosomil 5C_18_-AR-II HPLC column. Lycopaoctaene was purified by reversed phase HPLC using methanol:acetone (60:40) as a mobile phase at a flow rate of 3 ml min^−1^ and detection at 210 nm. Identity of lycopaoctaene molecule was confirmed by GC–MS (Supplementary Fig. 3) and NMR (Supplementary Table 1 and Supplementary Fig. 15).

### LSS and LOS complementation of yeast *SS* knockout strain

The yeast strain ZX 178-08 (*MAT***a**, *his3*, *leu2*, *met15*, *ura3*, *SUE* and *erg9Δ::HPH*), which has a knockout of the endogenous *SS* gene (*ERG9*), was used for complementation experiments^64^. The expression vector XURA used in our study was made by replacing the GAL1 and GAL10 promoters in pESC-URA vector backbone with constitutive promoters TEF1 and GPD, respectively^65^. Full-length *LSS* was cloned into XURA using Not1 and SpeI restriction sites. Full-length *LOS* was cloned into EcoRI and SpeI sites of XURA vector. The expression constructs *LSS*:XURA, *LOS*:XURA and XURA were transformed into ZX 178-08 yeast strain via lithium acetate transformation followed by selection on yeast synthetic dropout medium supplemented with ergosterol synthetic complete-Uracil (SCE URA). The positive transformants were further characterized by streaking on selection media with or without ergosterol, to test the ability of individual genes to restore ergosterol prototrophy of ZX 178-08 yeast.

### GC–MS analysis of PLOH

The enzyme assay for PLPP was conducted following the protocol described previously for analysis of PSPP^37^,^50^ and as follows. The PLPP assay contained 50 mM MOPS (pH 6.8), 2.5 mM β-mercaptoethanol, 20 mM MgCl_2_, 180 μM GGPP and 750 μg of purified LOS enzyme in a 2.5-ml total reaction volume. The reaction mixture was incubated at 37 °C for 2 h and terminated by snap freezing with liquid nitrogen followed by lyophilization. For hydrolysis of PLPP to PLOH, the white residue obtained after lyophilization was resuspended in 2 ml phosphatase solution (20% 1-propanol, 100 mM sodium acetate pH 4.7, 0.1% Triton-X-100 and 50 units of sweet potato acid phosphatase) and then incubated for 16 h in a 28 °C shaker. The dephosphorylation reaction mixture was extracted three times with 4 ml of methyl tertiary butyl ether (MTBE), the extracts pooled, dried under a stream of nitrogen gas, resuspended in 100 μl *n*-hexane and a 5-μl aliquot analysed by GC–MS as under the conditions described above with the following differences. Chemical ionization using methane gas at 20 psi was employed for analysis of the PLOH molecule, as electronic ionization did not result in a molecular ion of PLOH. Initial oven temperature was 70 °C, held for 1 min, then increased to 200 °C at the rate of 8 °C min^−1^ and then ramped to 300 °C at a rate of 20 °C min^−1^ and held for 20.75 min. Temperatures of injection port, interface and ion source were 260 °C, 250 °C and 200 °C, respectively.

## Additional information

**Accessions codes**: New DNA sequences described in this study have been deposited in GenBank: accession #KT388100, accession #KT388101 and accession #NM_001247787.

**How to cite this article:** Thapa, H. R. *et al*. A squalene synthase-like enzyme initiates production of tetraterpenoid hydrocarbons in *Botryococcus braunii* Race L. *Nat. Commun.* 7:11198 doi: 10.1038/ncomms11198 (2016).

## Supplementary Material

Supplementary Information Supplementary Figures 1-21 and Supplementary Table 1

## Figures and Tables

**Table 1:** LOS steady-state kinetic parameters for GGPP, FPP and PPP substrates.

	**GGPP**	**FPP**	**PPP**
*K*_m_ (mM)	0.07±0.02	0.13±0.02	0.11±0.01
*k*_cat_ (s^−1^)	1.14 × 10^−2^	2.05 × 10^−2^	1.16 × 10^−3^
*k*_cat_/*K*_m_ (μM^−1^ s^−1^)	1.65 × 10^−4^	1.62 × 10^−4^	1.03 × 10^−5^

FPP, farnesyl diphosphate, GGPP, geranylgeranyl diphosphate; LOS, lycopaoctaene synthase; PPP, phytyl diphosphate.

*K*_m_ values shown are the mean±s.e. obtained from three independent measurements (*n*=3).
