# Supplementary material for: A squalene synthase-like enzyme initiates production of tetraterpenoid hydrocarbons in Botryococcus braunii Race L
Source: Nat Commun. 2016 Apr 6;7:11198. doi: 10.1038/ncomms11198 (PMC4823828; doi:10.1038/ncomms11198)
Supplement: Supplementary Information — Supplementary Figures 1-21 and Supplementary Table 1 [file ncomms11198-s1.pdf]

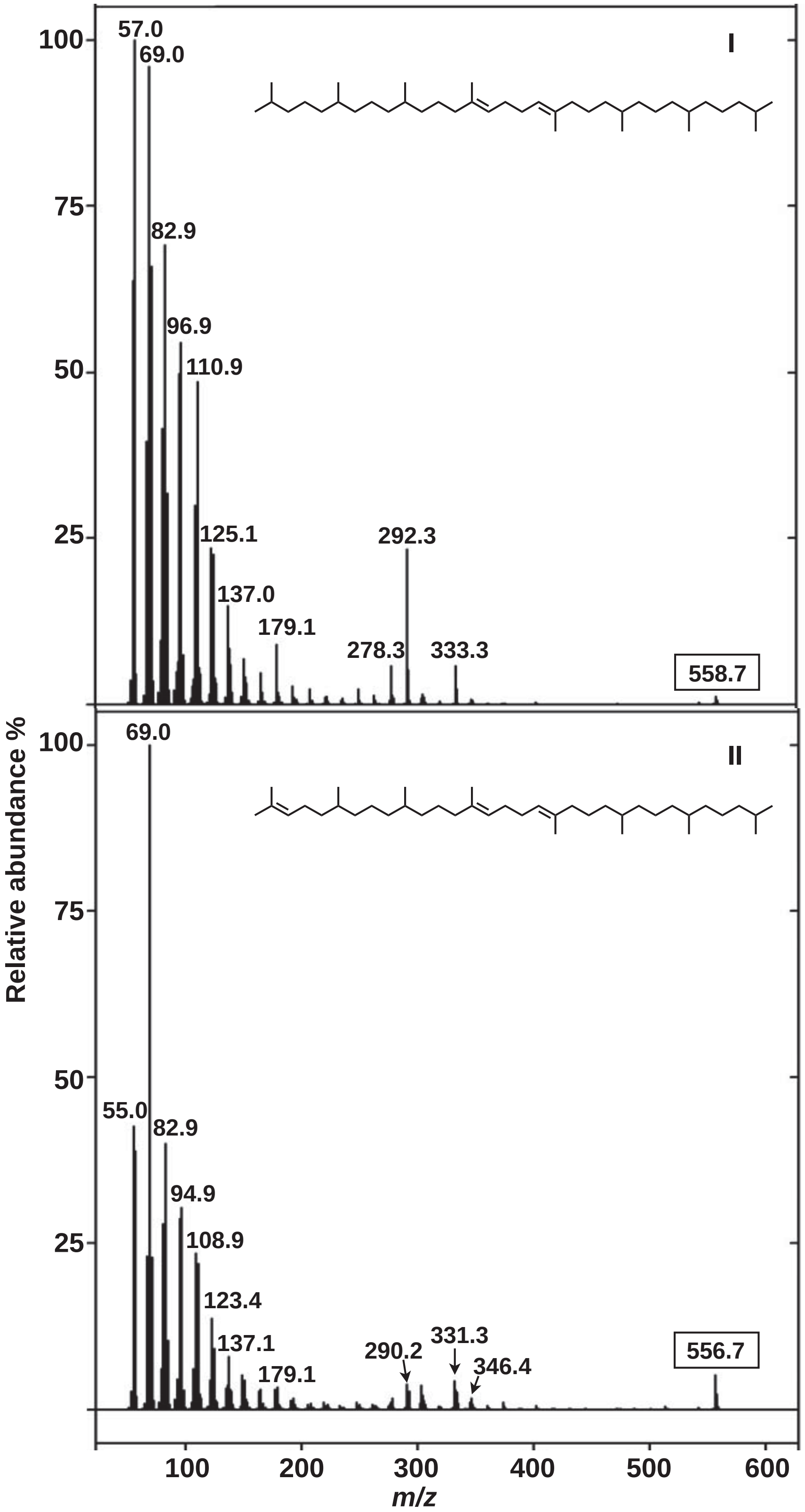

Supplementary Figure 1. The mass spectra from GC-MS and structures of lycopadiene and lycopatriene are shown in panel I and panel II, respectively. Molecular ion in box corresponds to the parent ion of each hydrocarbon molecule.

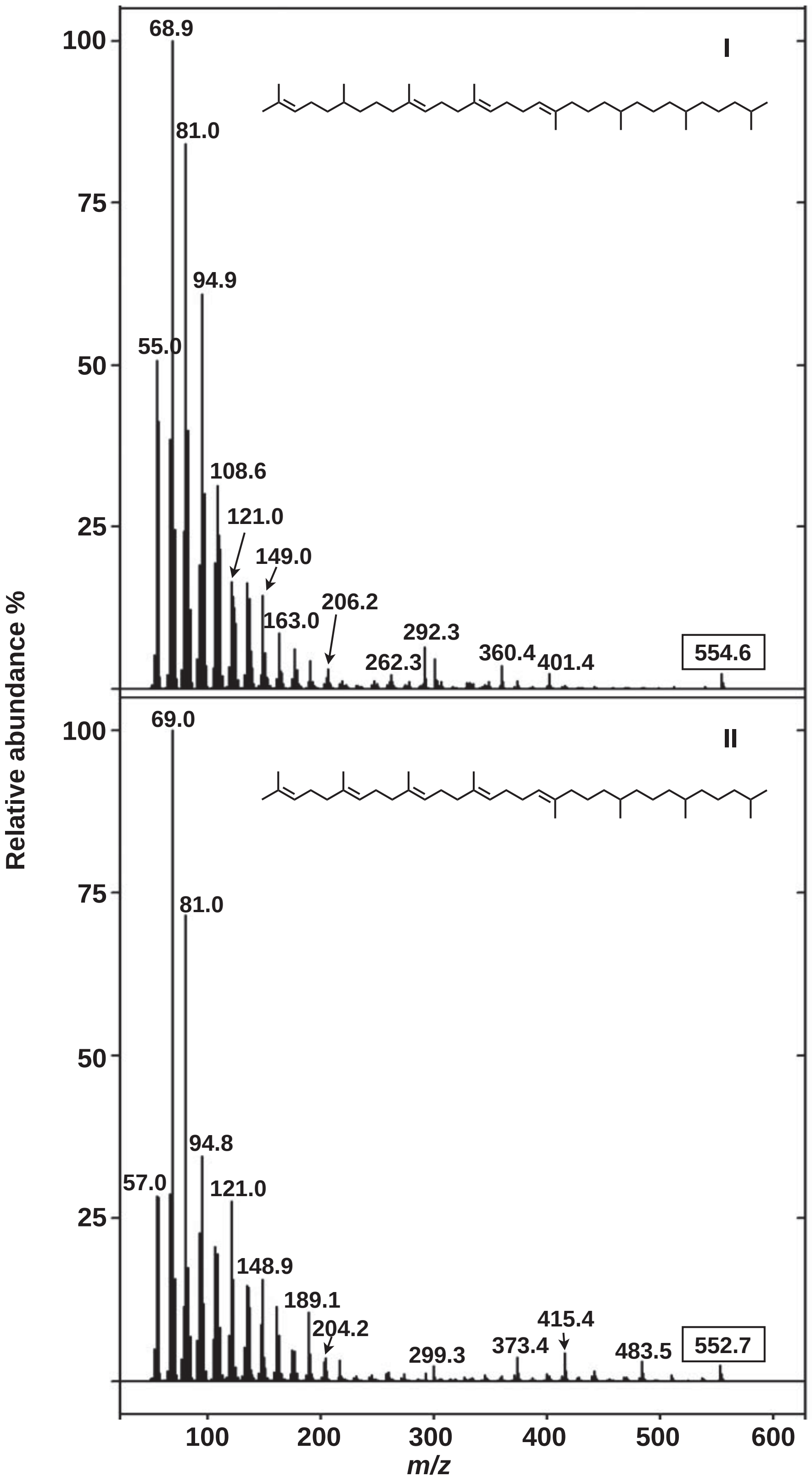

Supplementary Figure 2. The mass spectra from GC-MS and structures of lycopatetraene and lycopapentaene are shown in panel I and panel II, respectively. Molecular ion in box corresponds to the parent ion of each hydrocarbon molecule.

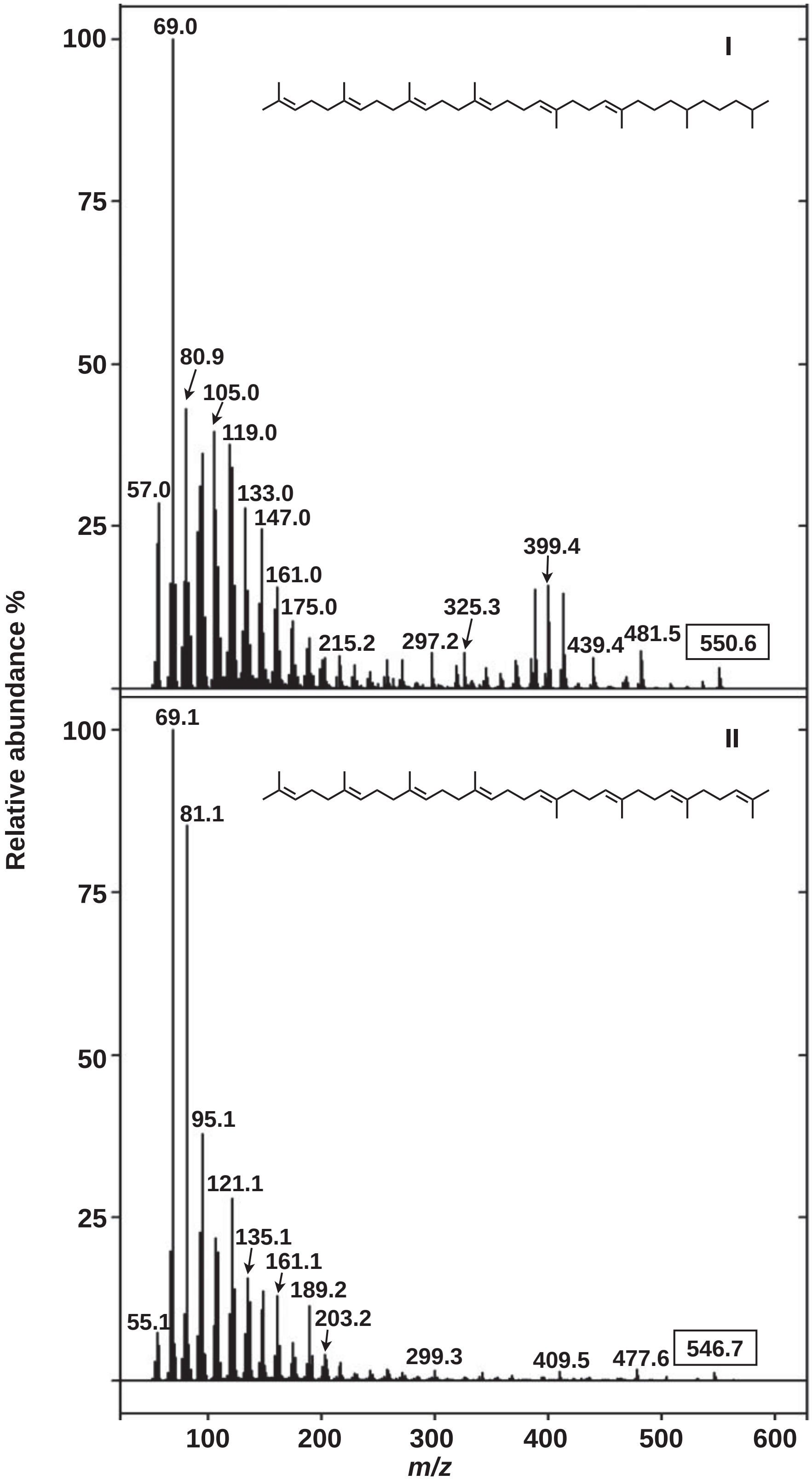

Supplementary Figure 3. The mass spectra from GC-MS and structures of lycopahexaene and lycopaoctaene are shown in panel I and panel II, respectively. Molecular ion in box corresponds to the parent ion of each hydrocarbon molecule.

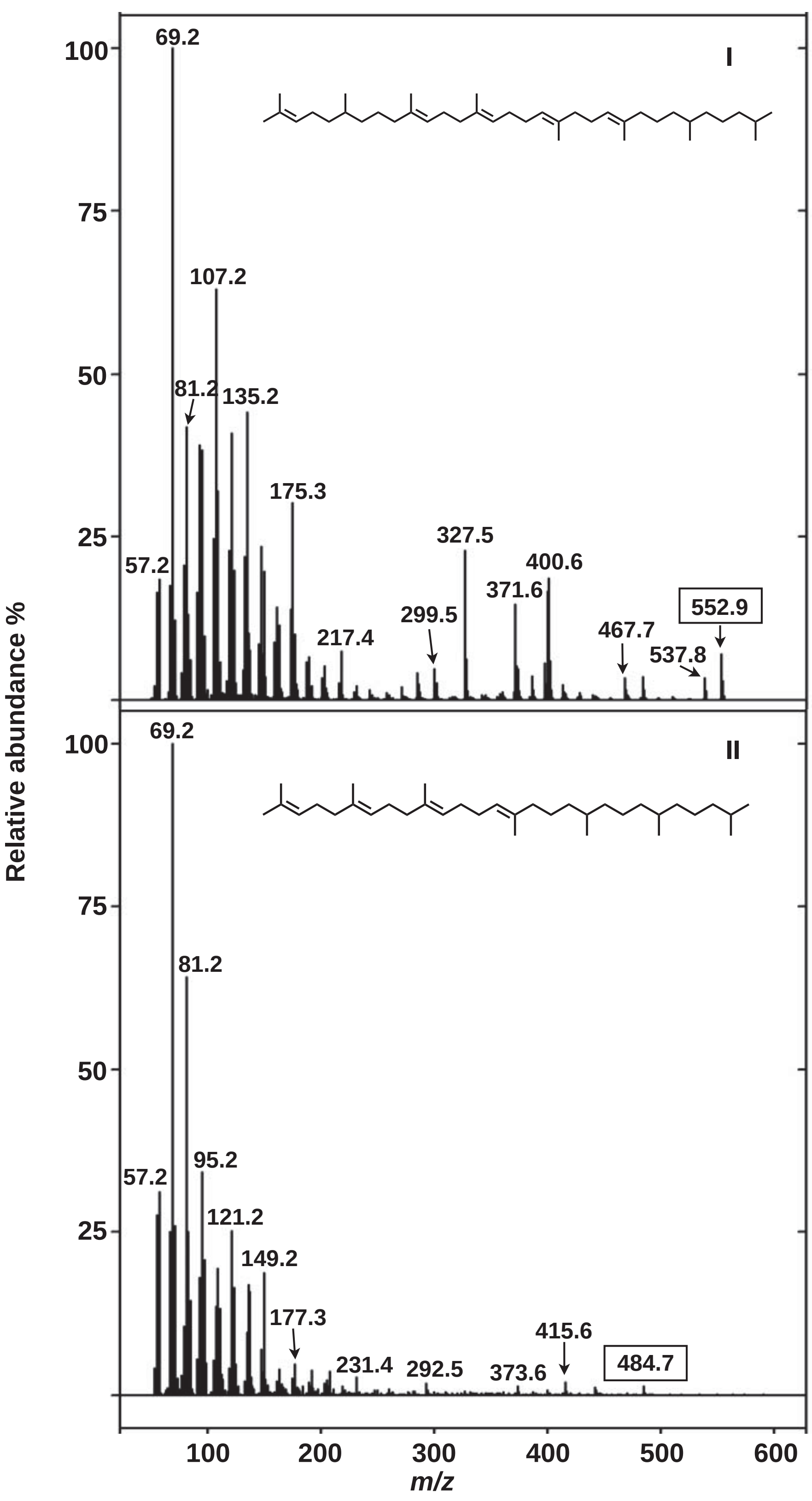

Supplementary Figure 4. The mass spectra from GC-MS and structures of lycopapentaene isomer and $C_{35}H_{64}$ are shown in panel I and panel II, respectively. Molecular ion in box corresponds to the parent ion of each hydrocarbon molecule.

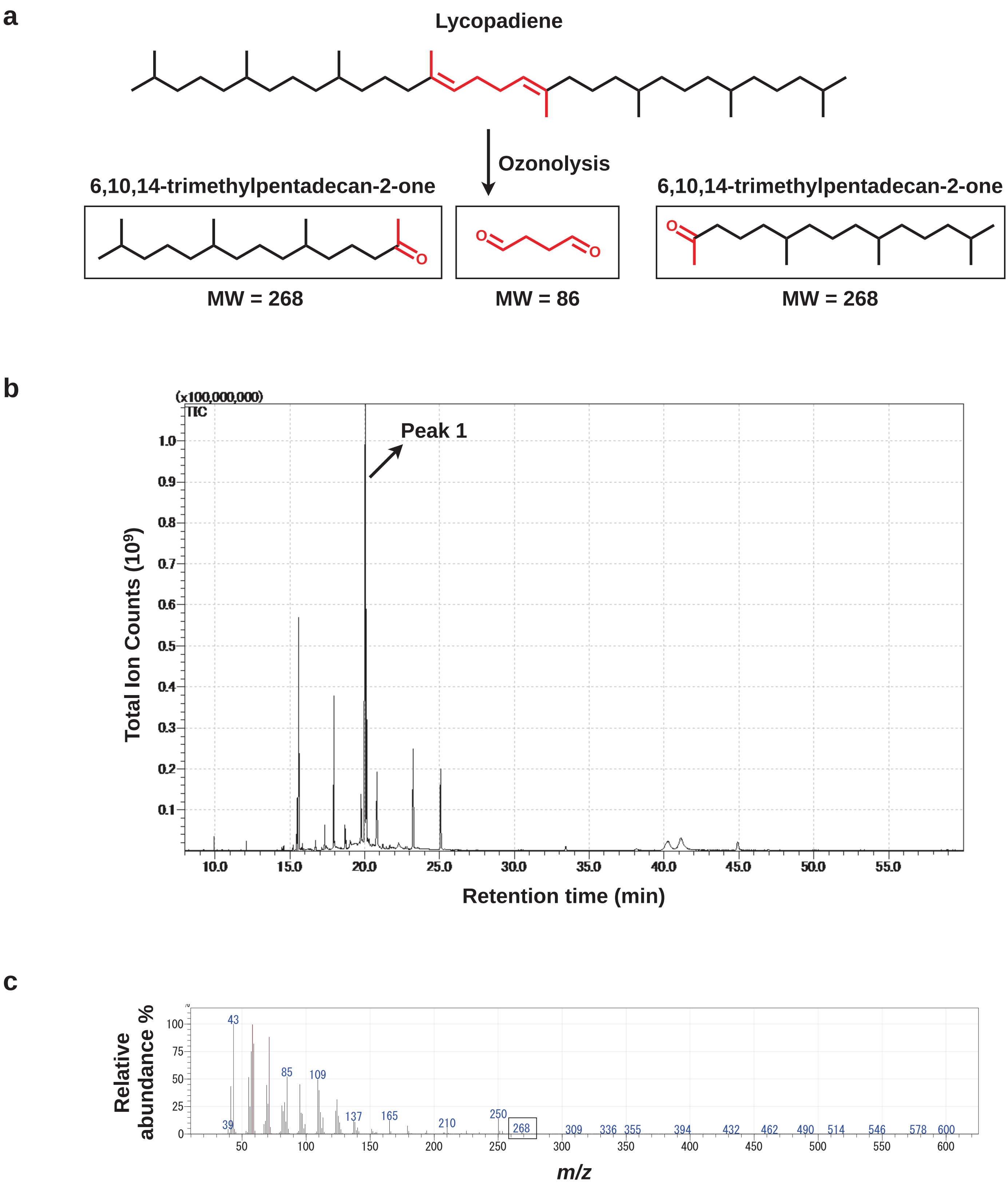

Supplementary Figure 5. **a**, Expected ozonolysis products from lycopadiene. **b**, GC-MS analysis of crude ozonolysis products from lycopadiene showed a predominant peak (Peak 1) at 20.04 min. **c**, The mass spectra of Peak 1 showed a parent ion of *m/z* = 268 (in box) and was identified as the expected molecule 6,10,14-trimethylpentadecan-2-one by the Shimadzu mass spectrum database.

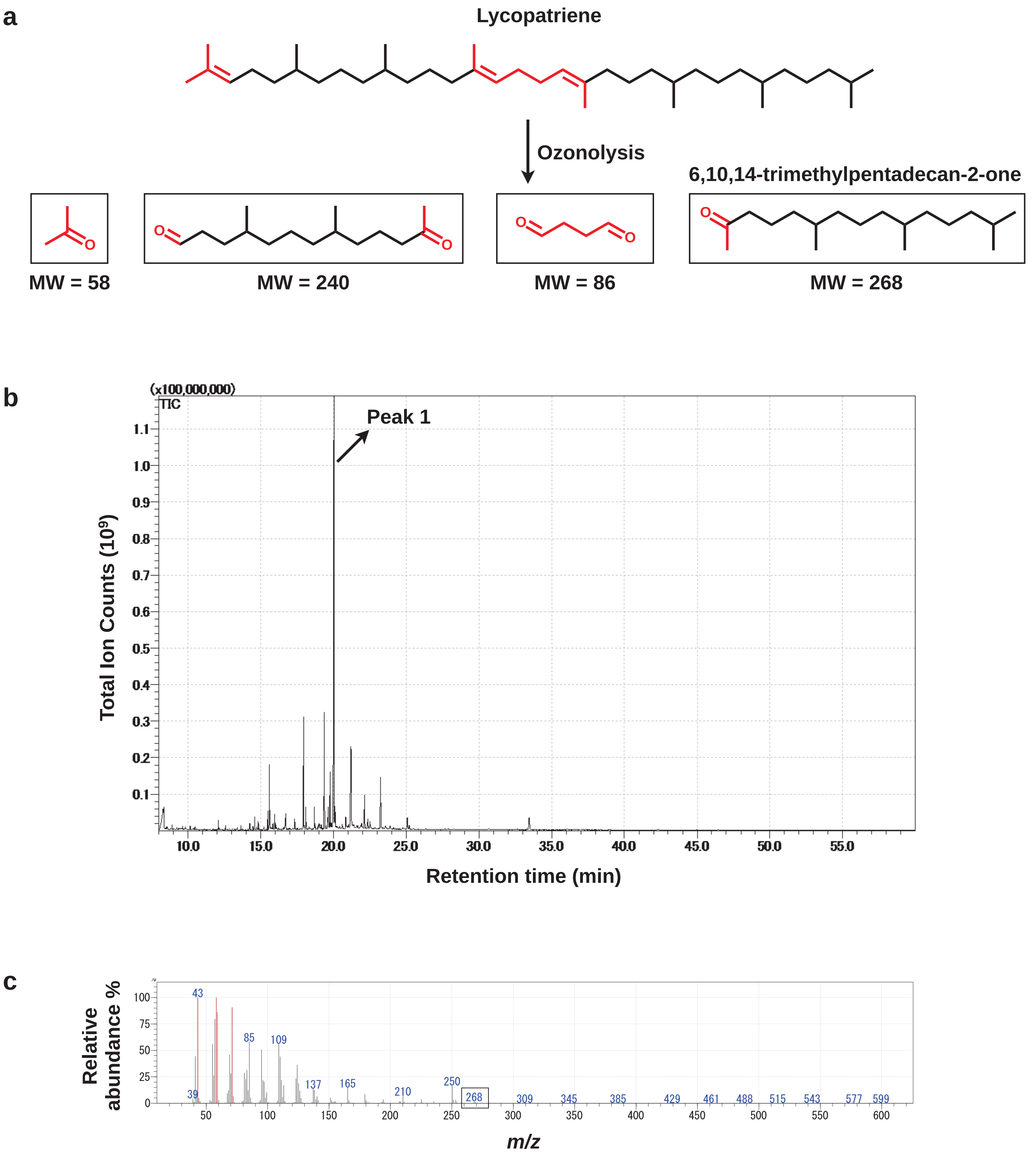

Supplementary Figure 6. **a**, Expected ozonolysis products from lycopatriene. **b**, GC-MS analysis of crude ozonolysis products from lycopatriene showed a predominant peak (Peak 1) at 20.04 min. **c**, The mass spectra of Peak 1 showed a parent ion of *m/z* = 268 (in box) and was identified as the expected molecule 6,10,14-trimethylpentadecan-2-one by the Shimadzu mass spectrum database.

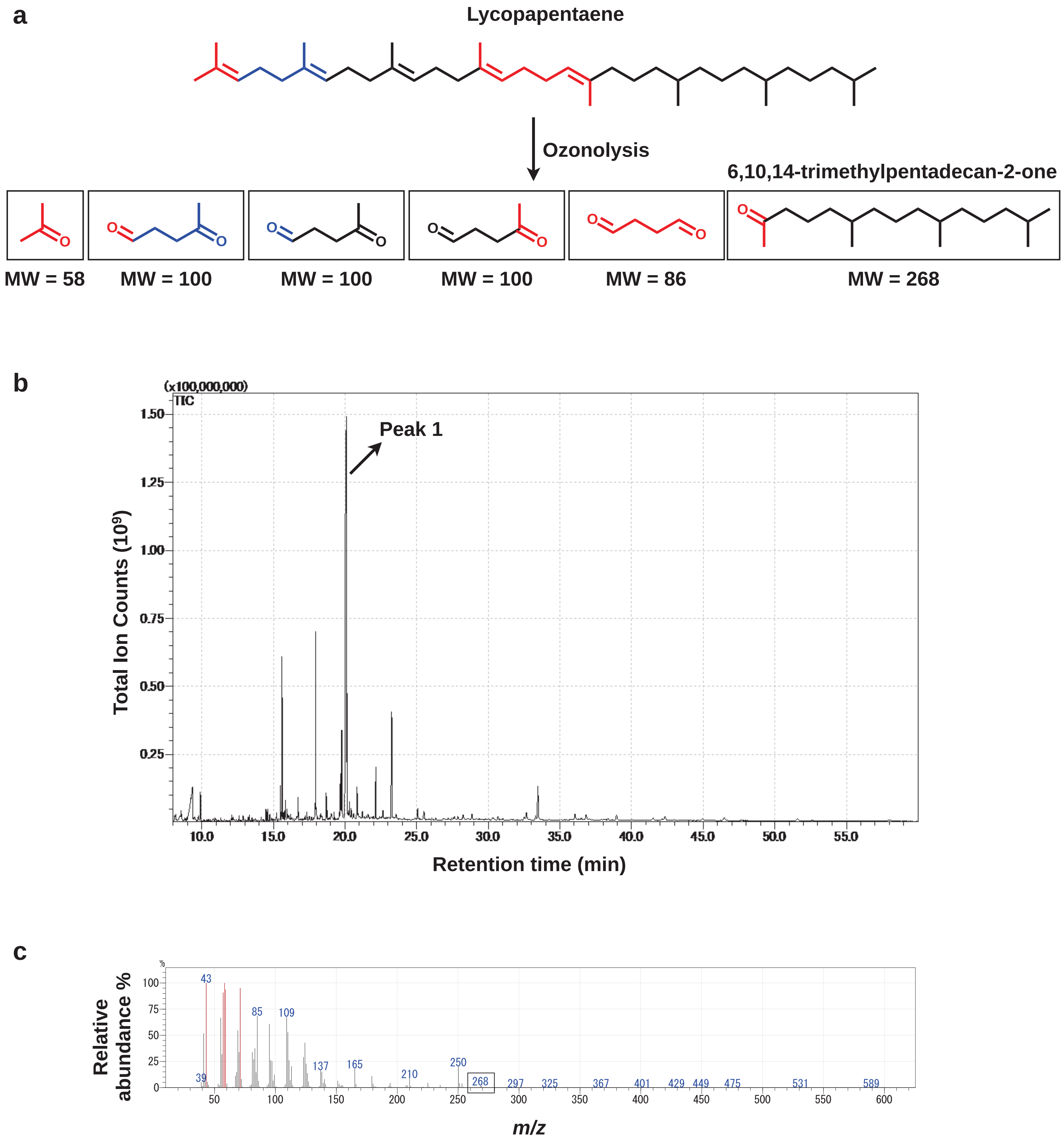

Supplementary Figure 7. **a**, Expected ozonolysis products from lycopapentaene. **b**, GC-MS analysis of crude ozonolysis products from lycopapentaene showed a predominant peak (Peak 1) at 20.04 min. **c**, The mass spectra of Peak 1 showed a parent ion of *m/z* = 268 (ion in box) and was identified as the expected molecule 6,10,14-trimethylpentadecan-2-one by the Shimadzu mass spectrum database.

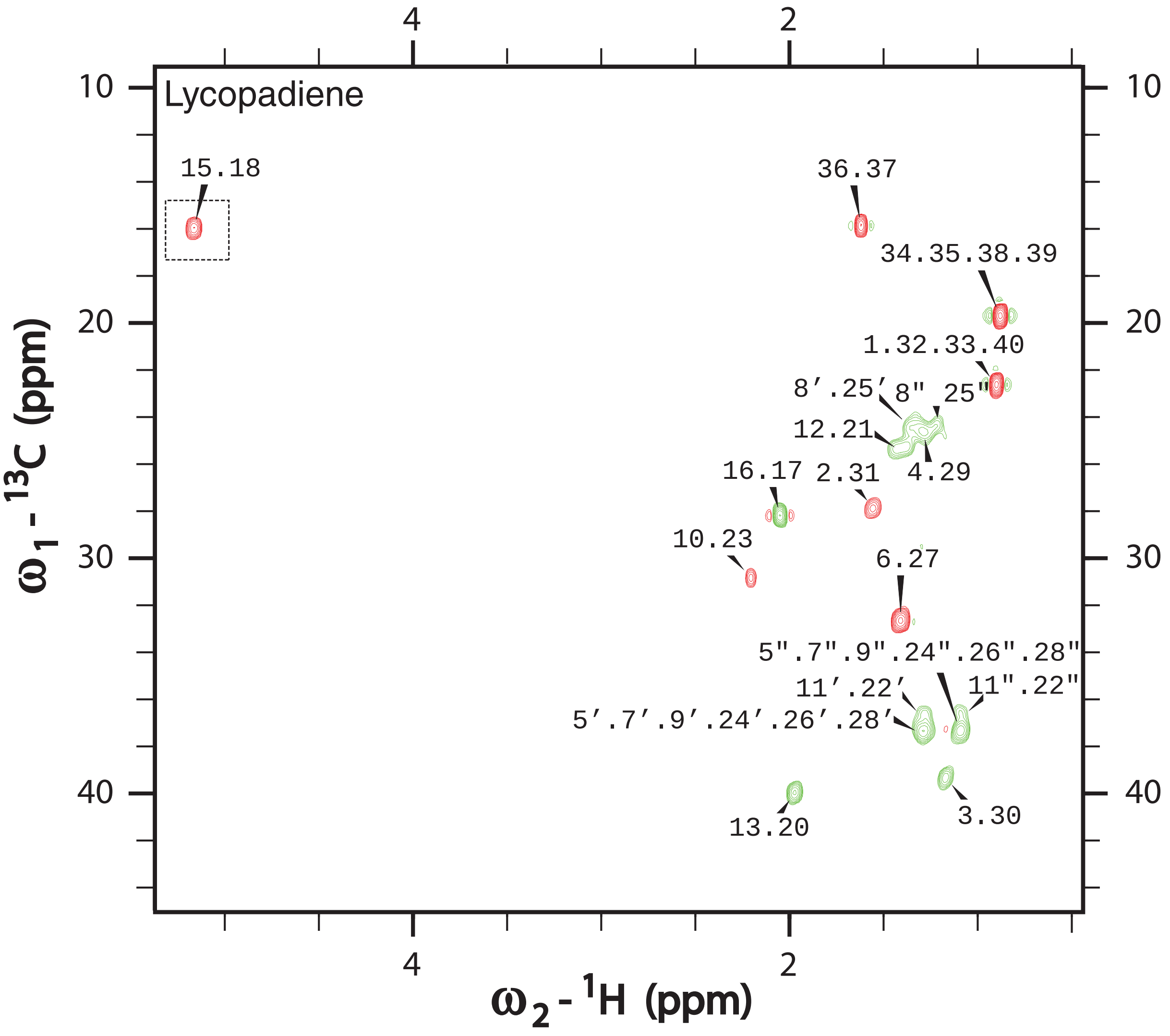

Supplementary Figure 8. 2D ^{13}C-HSQC spectrum of lycopadiene. Positive (red) contours represent -CH or -CH_3 correlations, while negative (green) contours indicate -CH_2. Peaks originating from the molecule of interest are marked with resonance assignment, while those from impurities are left unmarked. Peaks aliased on ^{13}C axis are shown in a dashed line box.

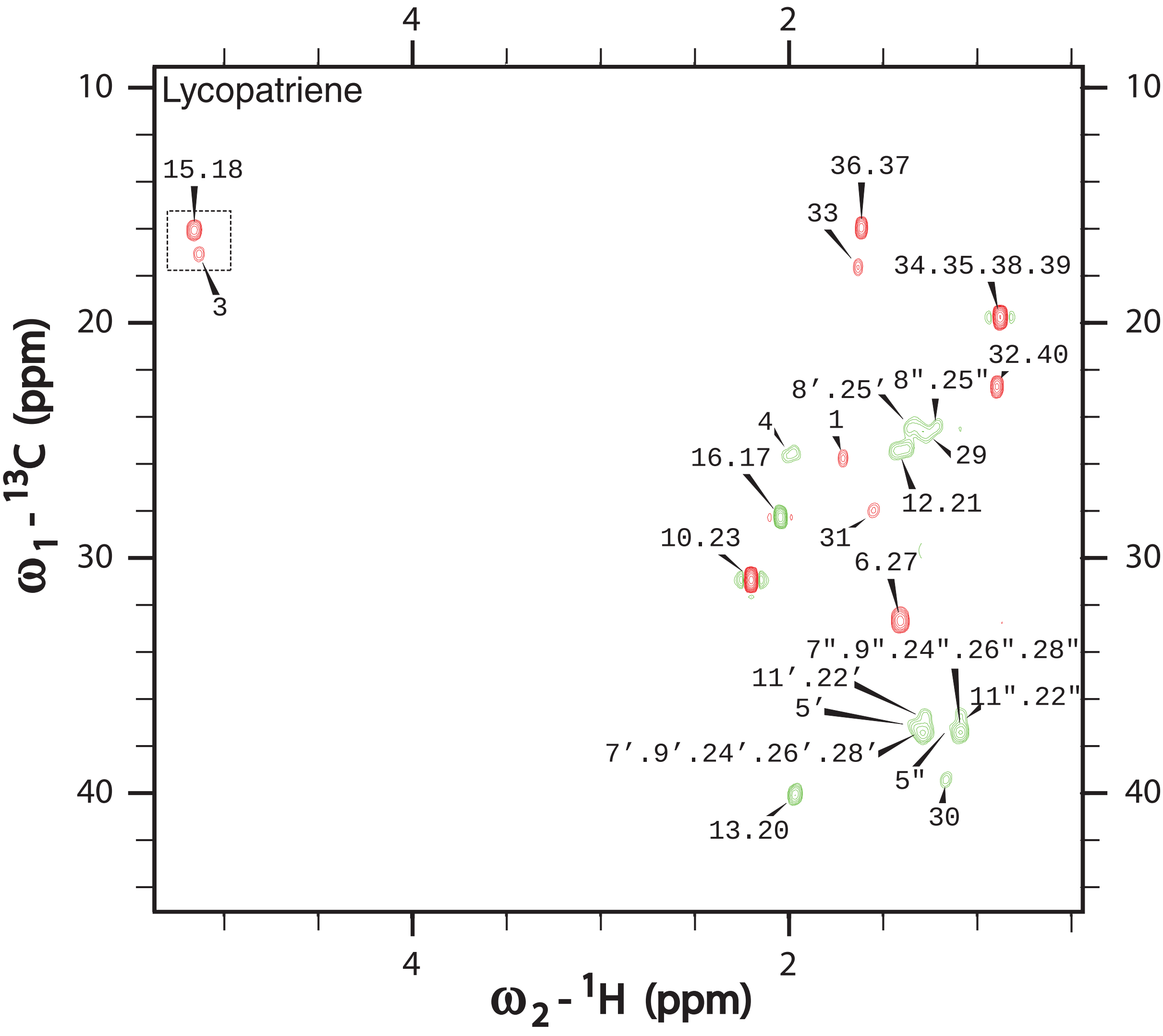

Supplementary Figure 9. 2D-^{13}C-HSQC spectrum of lycopatriene. Positive (red) contours represent -CH or -CH_3 correlations, while negative (green) contours indicate -CH_2. Peaks originating from the molecule of interest are marked with resonance assignment, while those from impurities are left unmarked. Peaks aliased on ^{13}C axis are shown in a dashed line box.

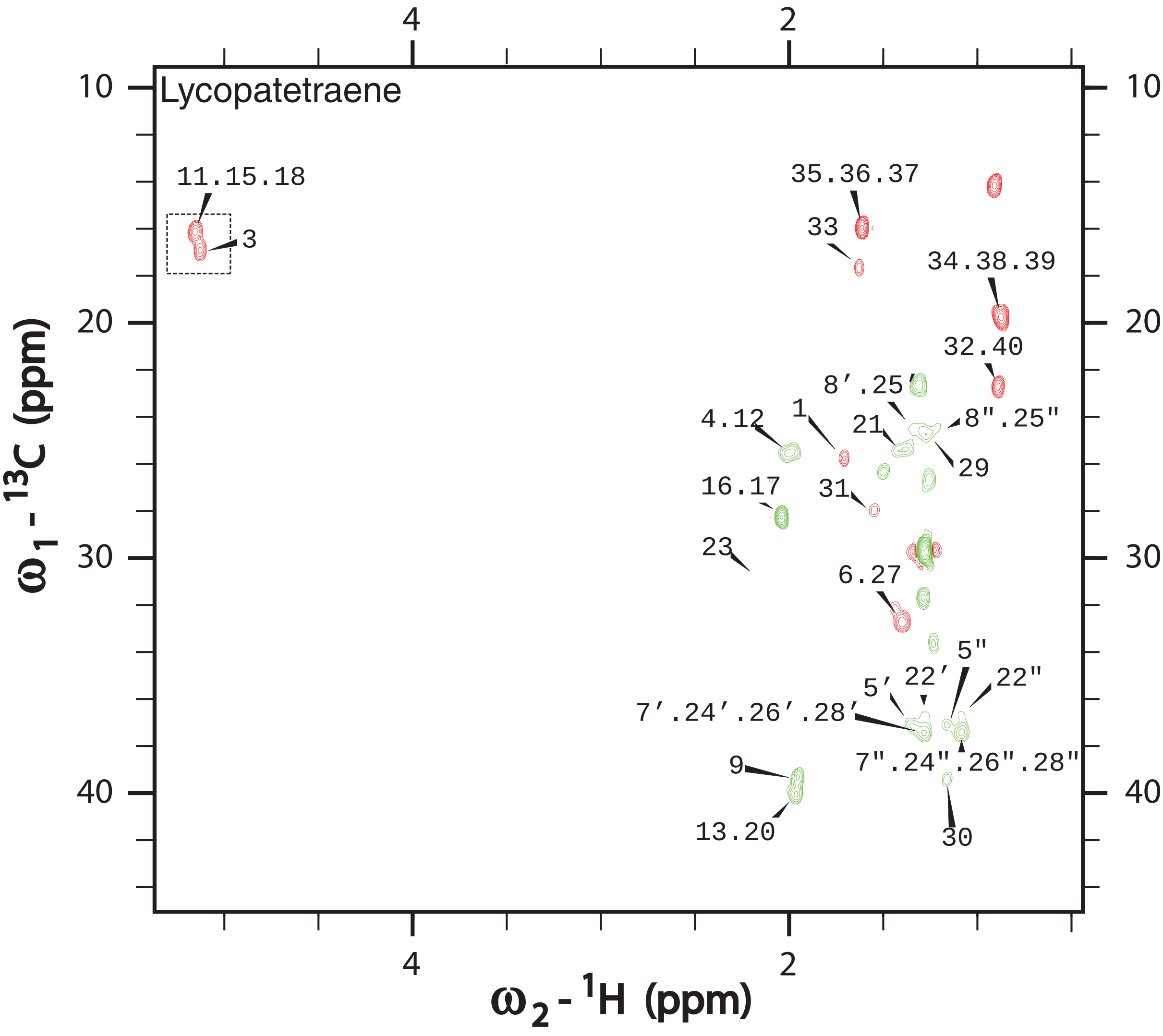

Supplementary Figure 10. 2D-^{13}C-HSQC spectrum of lycopatetraene. Positive (red) contours represent -CH or -CH_3 correlations, while negative (green) contours indicate -CH_2. Peaks originating from the molecule of interest are marked with resonance assignment, while those from impurities are left unmarked. Peaks aliased on ^{13}C axis are shown in a dashed line box.

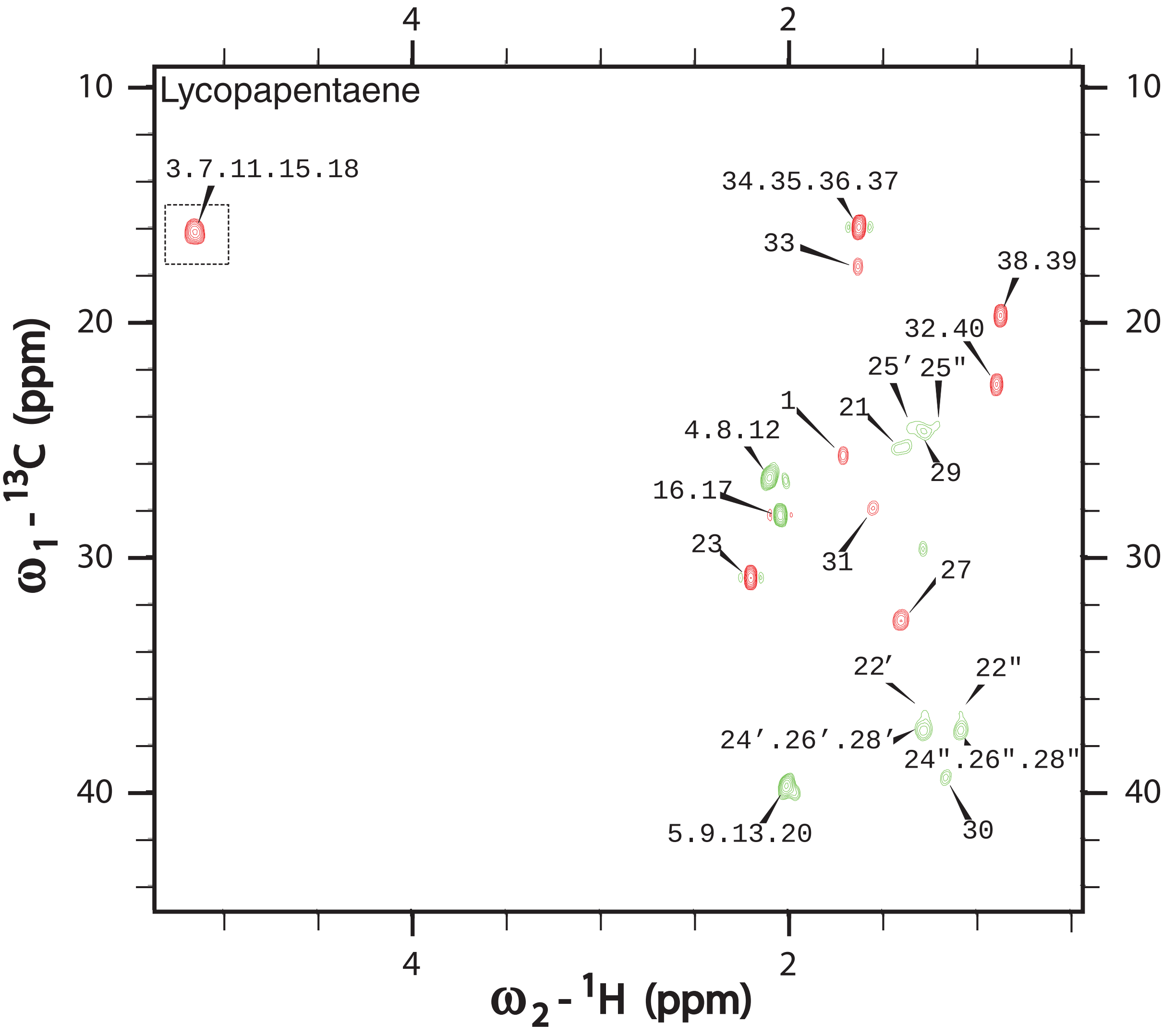

Supplementary Figure 11. 2D-^{13}C-HSQC spectrum of lycopapentaene. Positive (red) contours represent -CH or -CH_3 correlations, while negative (green) contours indicate -CH_2. Peaks originating from the molecule of interest are marked with resonance assignment, while those from impurities are left unmarked. Peaks aliased on ^{13}C axis are shown in a dashed line box.

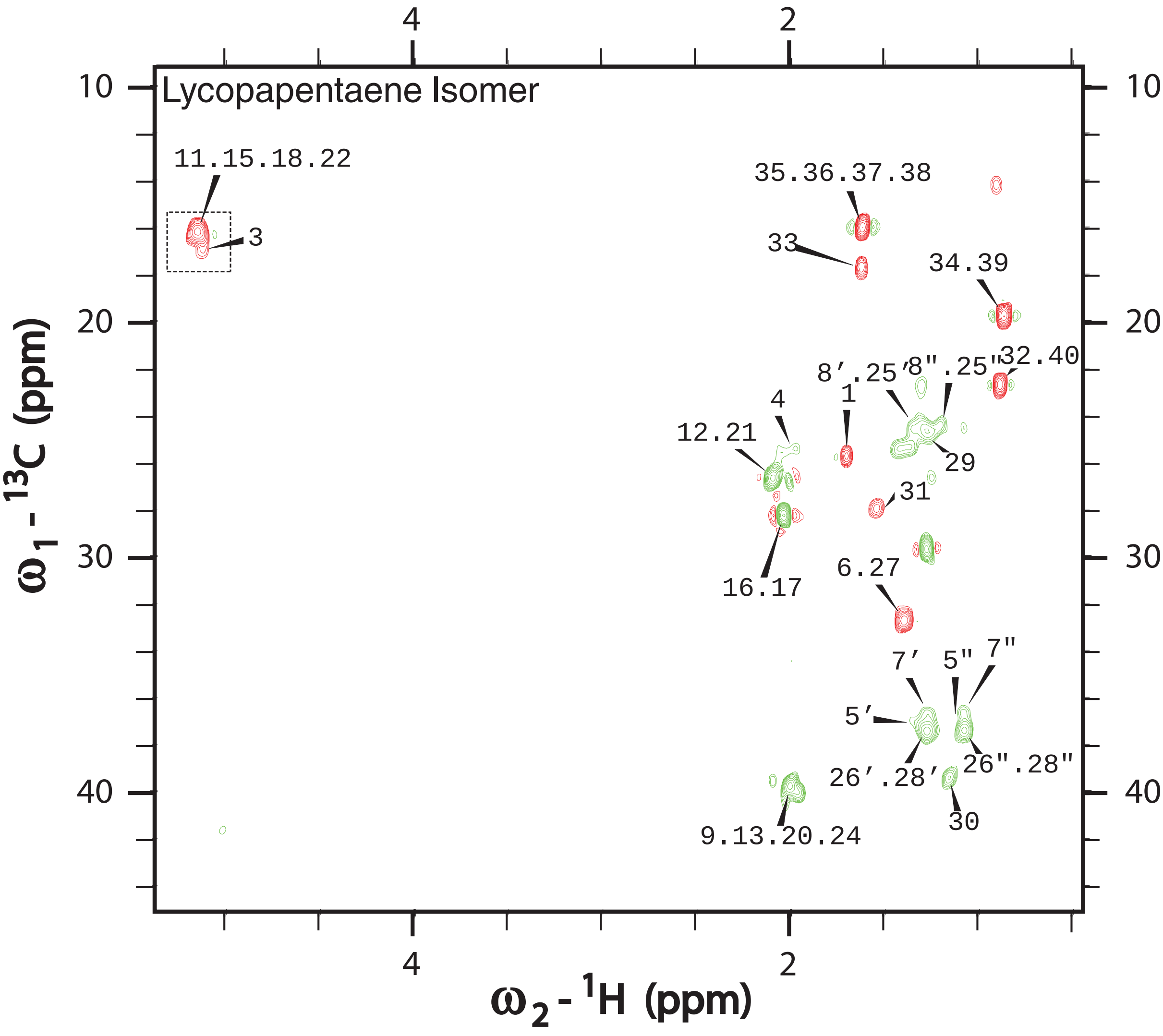

Supplementary Figure 12. 2D-^{13}C-HSQC spectrum of lycopapentaene isomer. Positive (red) contours represent -CH or -CH_3 correlations, while negative (green) contours indicate -CH_2. Peaks originating from the molecule of interest are marked with resonance assignment, while those from impurities are left unmarked. Peaks aliased on ^{13}C axis are shown in a dashed line box.

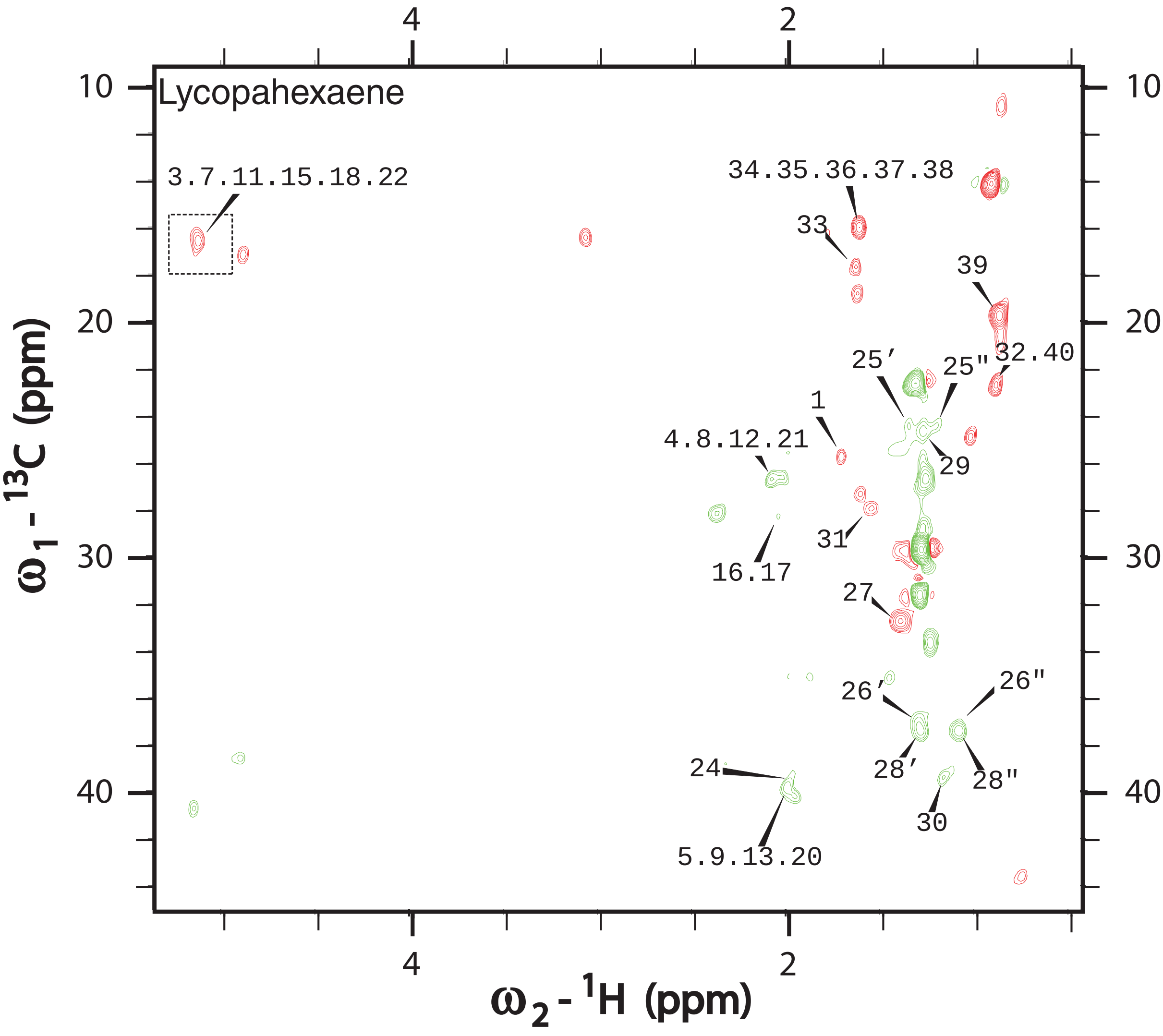

Supplementary Figure 13. 2D-^{13}C-HSQC spectrum of lycopahexaene. Positive (red) contours represent -CH or -CH_3 correlations, while negative (green) contours indicate -CH_2. Peaks originating from the molecule of interest are marked with resonance assignment, while those from impurities are left unmarked. Peaks aliased on ^{13}C axis are shown in a dashed line box.

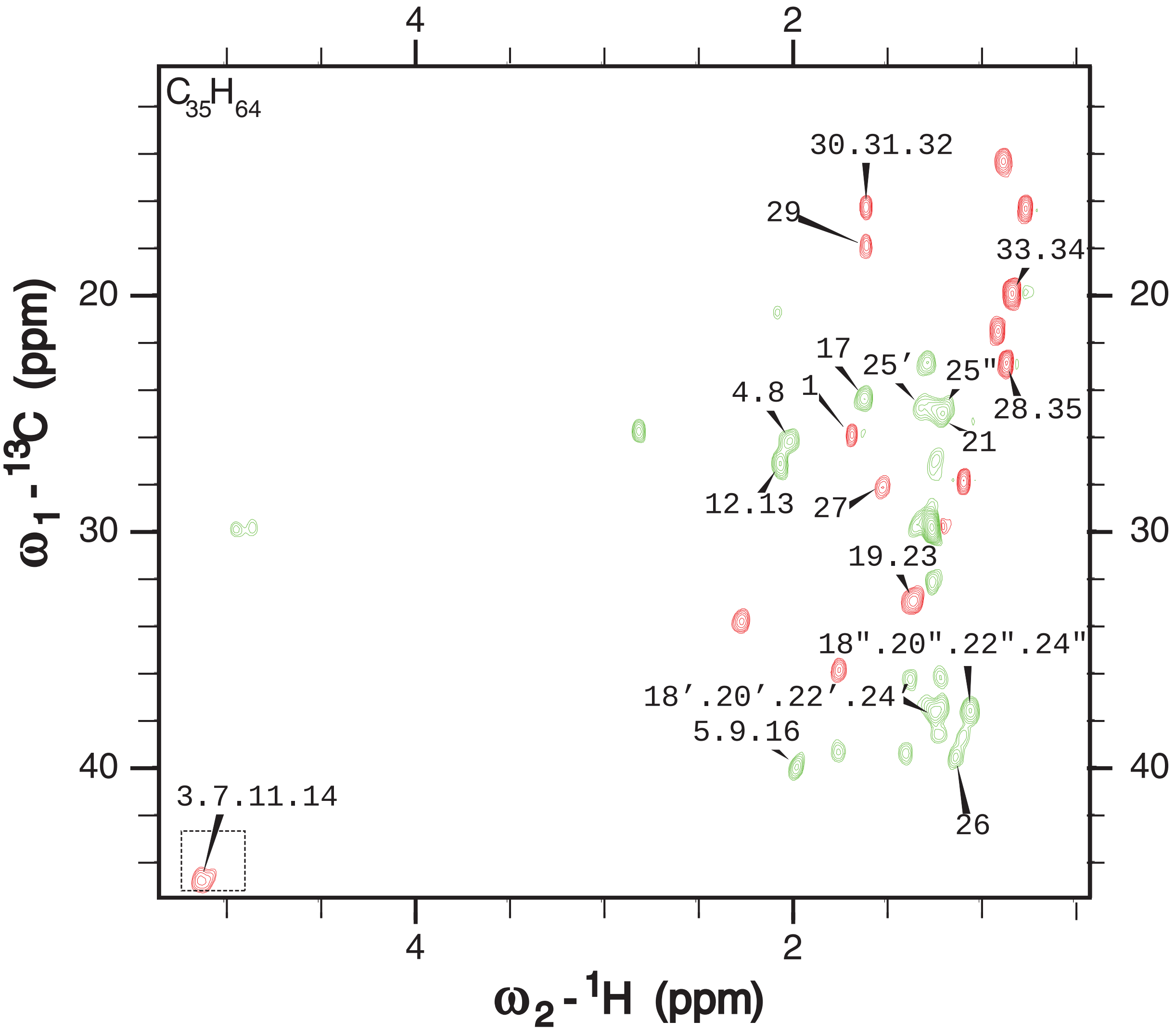

Supplementary Figure 14. 2D-^{13}C-HSQC spectrum of $C_{35}H_{64}$. Positive (red) contours represent -CH or -CH_3 correlations, while negative (green) contours indicate -CH_2. Peaks originating from the molecule of interest are marked with resonance assignment, while those from impurities are left unmarked. Peaks aliased on ^{13}C axis are shown in a dashed line box.

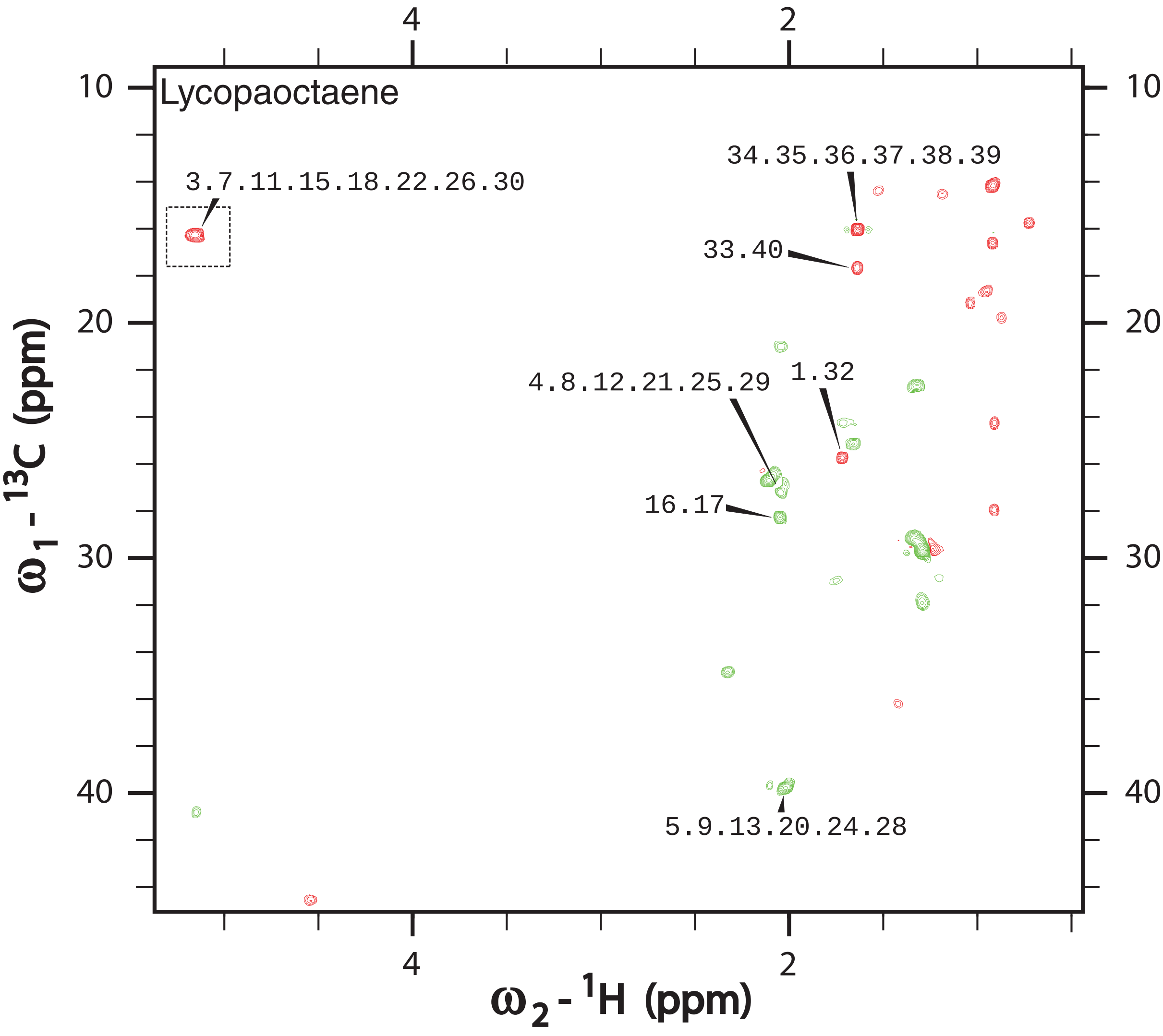

Supplementary Figure 15. 2D-^{13}C-HSQC spectrum of lycopaoctaene. Positive (red) contours represent -CH or -CH_3 correlations, while negative (green) contours indicate -CH_2. Peaks originating from the molecule of interest are marked with resonance assignment, while those from impurities are left unmarked. Peaks aliased on ^{13}C axis are shown in a dashed line box.

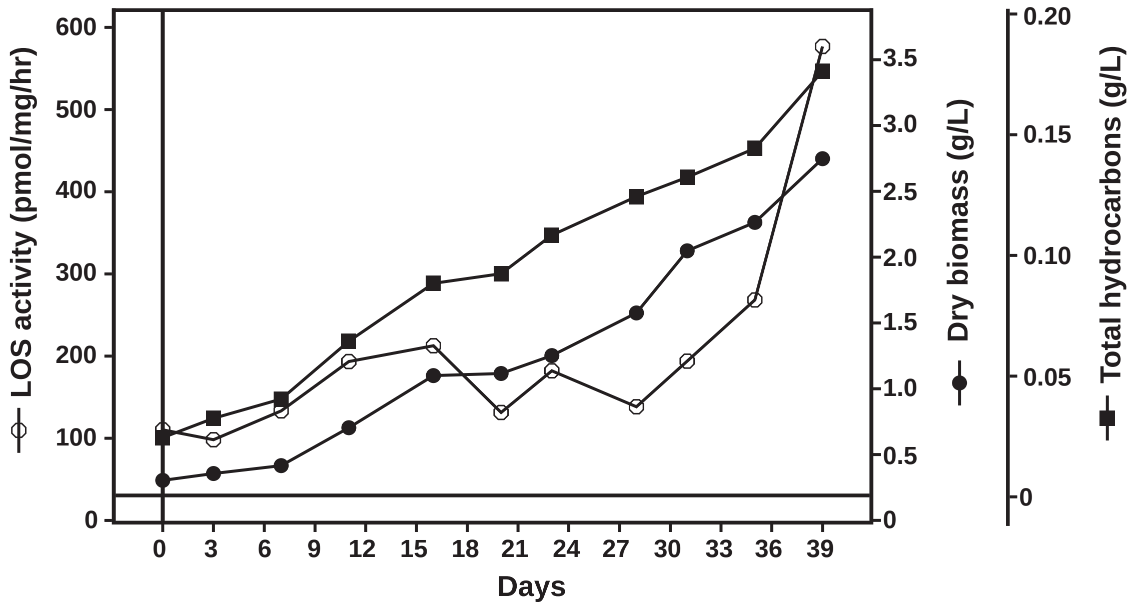

Supplementary Figure 16. Lycopaoctaene synthase (LOS) enzyme activity, growth rate (dry biomass accumulation), and total hydrocarbon production in race L over a 39-day culture period. Race L shows a rapid increase in growth rate after 12 days of inoculation into new medium, and a direct correlation between LOS enzyme activity and hydrocarbon accumulation was observed, with LOS activity increasing rapidly after day 30 of the culture cycle.

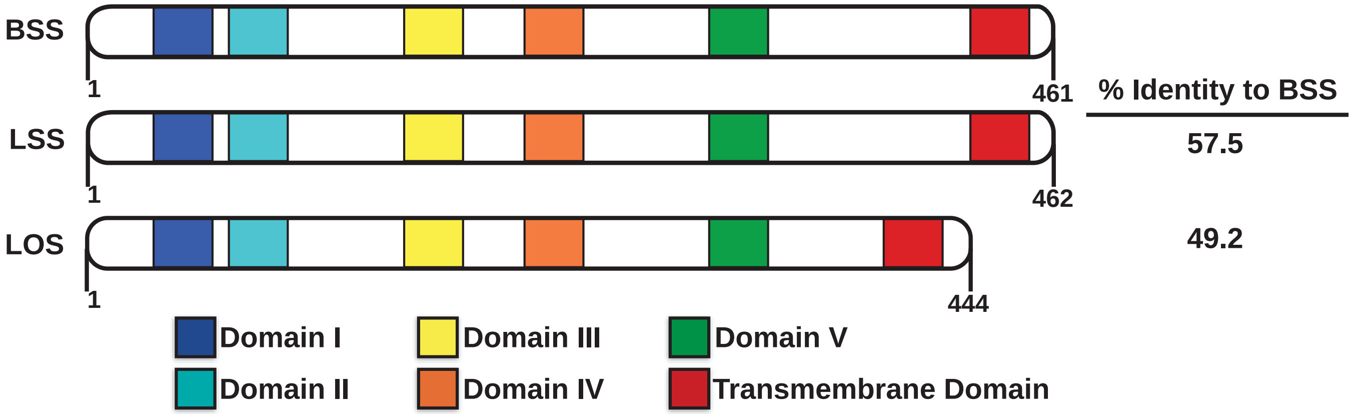

Supplementary Figure 17. Domain alignment of SS-like proteins from *B. braunii*. BSS = race B SS, LSS = race L SS, LOS = lycopaoctaene synthase.

```
                                                                         FLAP
LOS      -------MKYTDFLAHPDEIIPTIRMMYADYRLK----NMEIKDPSVRFCYNMLNRVSRSFAMVIQQ 56
LSS      ------MGKLQEVLKHPDELVPLMQMLVSDYYTK----I-VPRDPGLGFCYRMLNKVSRSFAIVIQQ 56
BSS      ---MGMLRWGVESLQNPDELIPVLRMIYADKFGK----I-KPKDEDRGFCYEILNLVSRSFAIVIQQ 59
SlSS     ------MGTLRAILKNPDDLYPLIKLKLAARHAE----KQIPPEPHWGFCYLMLQKVSRSFALVIQQ 57
SSL-1    MTMHQDHGVMKDLVKHPNEFPYLLQLAATTYGSPA---APIPKEPDRAFCYNTLHTVSKGFPRFVMR 64
SSL-2    ------MVKLVEVLQHPDEIVPILQMLHKTYRAK----R-SYKDPGLAFCYGMLQRVSRSFSVVIQQ 56
SSL-3    -------MKLREVLQHPGEIIPLLQMMVMAYRRK----R-KPQDPNLAWCWETLIKVSRSYVLVIQQ 55
HSS      -------MEFVKCLGHPEEFYNLVRFRIGGKRKVMPKMDQDSLSSSLKTCYKYLNQTSRSFAAVIQA 60
                      : .* ::   :::                 .     *:  *  .*:.:  .:
            Domain I        Domain II
LOS      LPVELRDATCVFYLILRALDTVEDDMAIPKEVKIPMLRTFHEHLSDRSWKIKCGY-GPYVDLMDNYP 122
LSS      LPELLRDPICVFYLVLRALDTVEDDMALPNDIKLPLLRAFHKKIYDRKWSMKCGY-GPYVQLMEEYP 122
BSS      LPAQLRDPVCIFYLVLRALDTVEDDMKIAATTKIPLLRDFYEKISDRSFRMTAGDQKDYIRLLDQYP 126
SlSS     LPVELRDAVCIFYLVLRALDTVEDDTSIPTDVKVPILISFHQHVYDREWHFACGT-KEYKVLMDQFH 123
SSL-1    LPQELQDPICIFYLLLRALDTVEDDMNLKSETKISLLRVFHEHCSDRNWSMKSDY-GIYADLMERFP 130
SSL-2    LPDELRHPICVFYLILRALDTVEDDMNLPNEVKIPLLRTFHEHLFDRSWKLKCGY-GPYVDLMENYP 122
SSL-3    LPEVLQDPICVNYLVLRGLDTLQDDMAIPAEKRVPLLLDYYNHIGDITWKPPCGY-GQYVELIEEYP 121
HSS      LDGEMRNAVCIFYLVLRALDTLEDDMTISVEKKVPLLHNFHSFLYQPDWRFMESK-EKDRQVLEDFP 126
         *   ::.  *: **:**.***::**  :    :: :*  ::.   :  :            ::: :
                                                               Domain III
LOS      LVTDVYLRFDEGTKAVIKDITRRMGNGMADFIDLDEVLTIPQYDLYCHYVAGLCGIGMCKLFVDSGL 189
LSS      MVTGVFLKLDPGPREVITEICRKMGAGMAEFIPK-EVLTVKDYDQYCHYAAGLVGEGLSKLAVGSGL 188
BSS      KVTSVFLKLTPREQEIIADITKRMGNGMADFVHKGVPDTVGDYDLYCHYVAGVVGLGLSQLFVASGL 193
SlSS     HVSTAFLELGKLYQQAIEDITMRMGAGMAKFICK-EVETTDDYDEYCHYVAGLVGLGLSKLFHASGK 189
SSL-1    LVVSVLEKLPPATQQTFRENVKYMGNGMADFIDK-QILTVDEYDLYCHYVAGSCGIAVTKVIVQFNL 196
SSL-2    LVTDVFLTLSPGAQEVIRDSTRRMGNGMADFIGKDEVHSVAEYDLYCHYVAGLVGSAVAKIFVDSGL 189
SSL-3    RVTKEFLKLNKQDQQFITDMCMRLGAEMTVFLKR-DVLTVPDLDLYAFTNNGPVAICLTKLWVDRKF 187
HSS      TISLEFRNLAEKYQTVIADICRRMGIGMAEFLDK-HVTSEQEWDKYCHYVAGLVGIGLSRLFSASEF 192
          :      :    :  : :   :*  *: *:       :  : * *..   *  .  : ::
                   Domain IV
LOS      EKEDLVAEEDLANQMGLFLQKNNIVRDYLEDINELPAPRMFWPKEIWGNYAKQLDEFKDPKNLDKAM 256
LSS      ENPVLLQKEDLSNHMGLFLQKTNIVRDYLEDINEEPAPRMFWPKEIWGKYTKDLADFKDPANEKGAV 255
BSS      QSPSLTRSEDLSNHMGLFLQKTNIIRDYFEDINELPAPRMFWPREIWGKYANNLAEFKDPANKAAAM 260
SlSS     ED---LASDSLSNSMGLFLQKTNIIRDYLEDINEVPKCRMFWPREIWSKYVNKLEDLKYEENSVKAV 253
SSL-1    ATPE-ADSYDFSNSLGLLLQKANIITDYNEDINEEPRPRMFWPQEIWGKYAEKLADFNEPENIDTAV 262
SSL-2    EKENLVAEVDLANNMGQFLQKTNVIRDYLEDINEEPAPRMFWPREIWGKYAQELADFKDPANEKAAV 256
SSL-3    ADPKLLDREDLSGHMAMFLGKINVIRDIKEDVLEDP-PRIWWPKEIWGKYLKDLRDIIKPEYQKEAL 253
HSS      EDPLVGEDTERANSMGLFLQKTNIIRDYLEDQQ---GGREFWPQEVWSRYVKKLGDFAKPENIDLAV 256
                  . :  :. :* *::* **       * :**:*:*..* :.* ::        *:
                                         Domain V                JK loop
LOS      LCLNHMVTDALRHCEVGLRSLSLLHNPNILRAVLIPQVMGVRTLTLVYNNPEVFRG---VVKMRRGE 320
LSS      QCLNHMVTDALRHGEHALKYMALLRDPQYFNFCAIPQVMAFGTLSLCYNNPQVFKG---VVKLRKGE 319
BSS      CCLNEMVTDALRHAVYCLQYMSMIEDPQIFNFCAIPQTMAFGTLSLCYNNYTIFTGPKAAVKLRRGT 327
SlSS     QCLNDMVTNALSHVEDCLTYMFNLHDPAIFRFCAIPQVMAIGTLAMCYDNIEVFRG---VVKMRRGL 317
SSL-1    KCLNHMVTDAMRHIEPSLKGMVYFTDKTVFRALALLLVTAFGHLSTLYNNPNVFKE---KVRQRKGR 326
SSL-2    QCLNHMVTDALRHCEIGLNVIPLLQNIGILRSCLIPEVMGLRTLTLCYNNPQVFRG---VVKMRRGE 320
SSL-3    ACLNDILTDALRHIEPCLQYMEMVWDEGVFKFCAVPELMSLATISVCYNNPKVFTG---VVKMRRGE 317
HSS      QCLNELITNALHHIPDVITYLSRLRNQSVFNFCAIPQVMAIATLAACYNNQQVFKG---AVKIRKGQ 320
          ***.::*:*: *    :  :  . :   :.   :    ..  ::  *:*  :*      *:  *:*

LOS      TAKIFVTTTSKLSFFRTYLQFANEMEQKCLTEAKNDPMVALTLKRVQGVQAACRAAIVKAEIAE--G 385
LSS      SAKLMTTVKSMPALYRTFLRMADDMVARCKGEARQDPNVATTLKRLQAIQAVCKTGLRSSIKSRKKQ 386
BSS      TAKLMYTSNNMFAMYRHFLNFAEKLEVRCNTETSEDPSVTTTLEHLHKIKAACKAGLARTKDDT--- 391
SlSS     TAKVIDRTKTMADVYGAFFDFSCMLKSKVNN---NDPNATKTLKRLDAILKTCRDSGTLNKRKSYII 381
SSL-1    IARLVMSSRNVPGLFRTCLKLANNFESRCKQETANDPTVAMTIKRLQSIQATCRDGLAKYDTPSGLK 393
SSL-2    TAKLFMSIYDKRSFYQTYLRLANELEAKCKGEASGDPMVATTLKHVHGIQKSCKAALSSKELLA--K 385
SSL-3    TAKLFLSVTNMPALYKSFSAIAEEMEAKCVR---EDPNFALTVKRLQDVQALCKAGLAKSNGKVSAK 381
HSS      AVTLMMDATNMPAVKAIIYQYMEEIYHRIPD---SDPSSSKTRQIISTIRTQNLPNCQ--------L 376
          . :.        .          :  :      **  : * : :  :

LOS      AKGP-STAMVLAGALLIAALAYFAYVYSAGGTSLKA-LPLFG--VVIILAIGLFGRNLALKTV---- 444
LSS      AATPLSDDFI-SKLVLVLGLGYCVYAFNLLPLLWKSALIPGPPPPALTSSLGLPHQIIAVFCVLTAG 452
BSS      -----FDELRSRLLALTGGSFYLAWTYNFLDLRGPGDLPTFLSVTQHWWSILIFLISIAV------- 446
SlSS     RNEPNYSPVLIVVI-------FIILAIILAQLFGSRS------------------------------ 411
SSL-1    SFCAAPTPTK--------------------------------------------------------- 403
SSL-2    SGSALTDDPAIRLLLLVGVVAYFAYAFNLGDVRGEHGVRALG--SILD--L--SQKGLAVASVALLL 446
SSL-3    GA----------------------------------------------------------------- 383
HSS      ISRSHYSPIYLSFVMLLAAL---SWQYLTTLSQVTEDYVQTGEH----------------------- 417

LOS      ------------------- 444
LSS      YQVFLRGGLA--------- 462
BSS      --FFIPSRPSPRPTLSA-- 461
SlSS     ------------------- 411
SSL-1    ------------------- 403
SSL-2    LVLLARSRLPLLTSASSKQ 457
SSL-3    ------------------- 383
HSS      ------------------- 417
```

Protein	% identity to LOS
LSS	52.5
BSS	49.2
*Sl*SS	47.3
SSL-1	44.6
SSL-2	61.7
SSL-3	44.1
HSS	33.9

Supplementary Figure 18. Amino acid sequence alignment and percent identity of SS and SS-like proteins using Clustal Omega. FLAP region, five conserved domains and putative NADPH binding residues (indicated in red) found in typical squalene synthase are labeled and shown in bold. Possible transmembrane region(s) predicted by TMpred for each protein are underlined. HSS = human squalene synthase, LOS = lycopaoctaene synthase, LSS = race L SS, BSS = race B SS, *Sl*SS = SS from *Solanum lycopersicum*, and SSL-1, SSL-2, SSL-3, SS-like enzymes from race B.

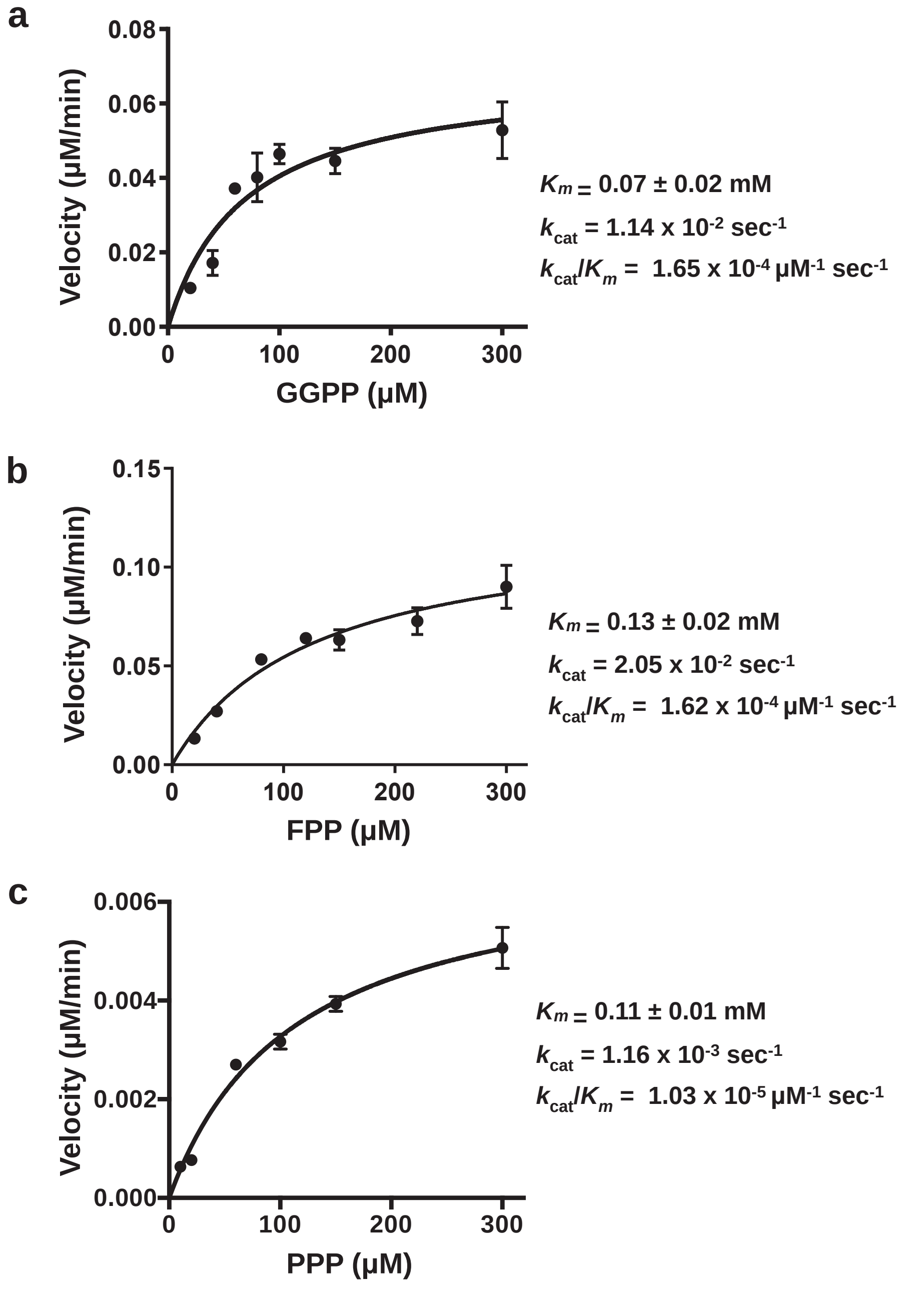

Supplementary Figure 19. Michaelis–Menten enzyme kinetics of the LOS reaction with different substrates. **a**, LOS kinetics using GGPP as substrate. **b**, LOS kinetics using FPP as substrate. **c**, LOS kinetics using phytyl-PP as substrate.

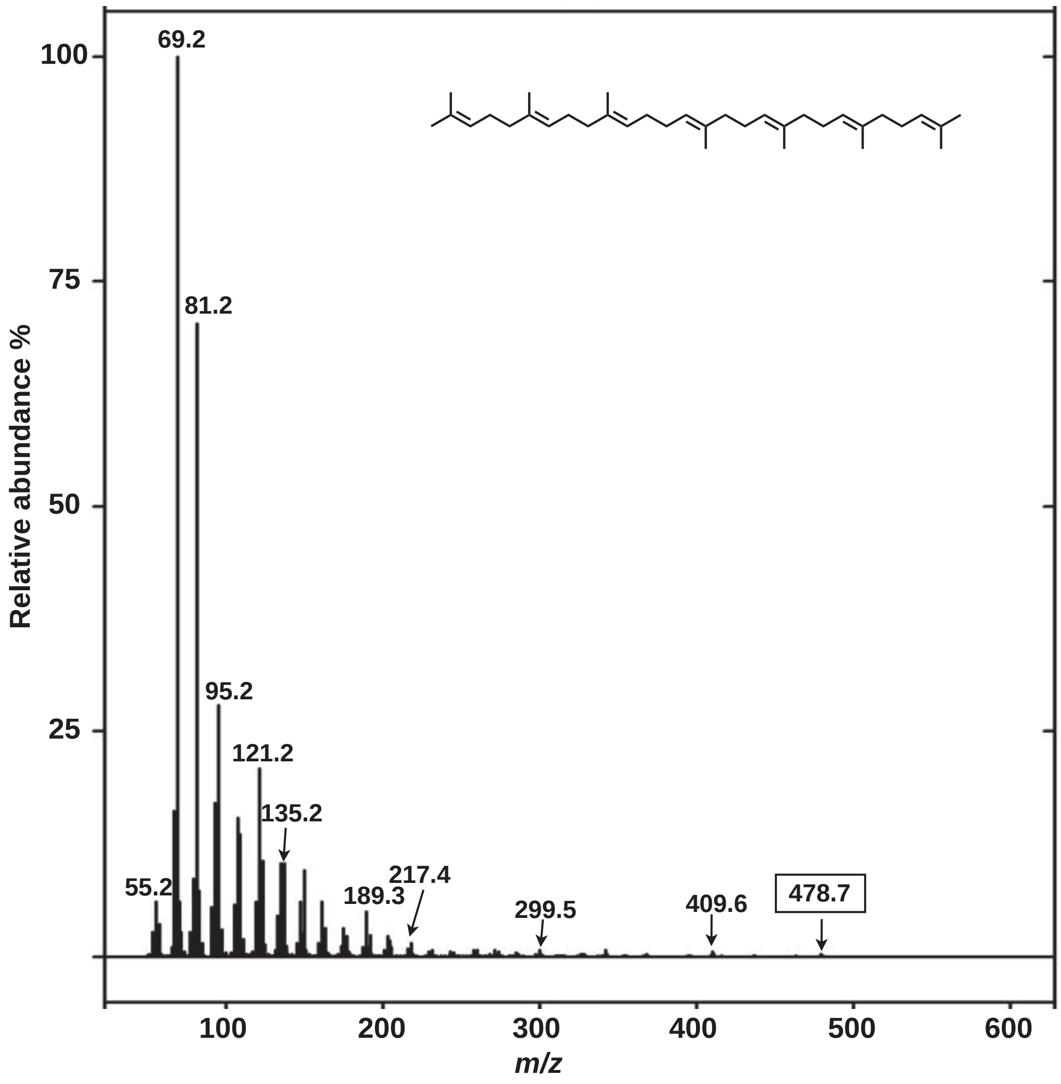

Supplementary Figure 20. The mass spectrum and structure of the $C_{35}H_{58}$ molecule produced by the LOS enzyme when FPP and GGPP are used in combination as substrate. Molecular ion in box corresponds to the parent ion of the molecule.

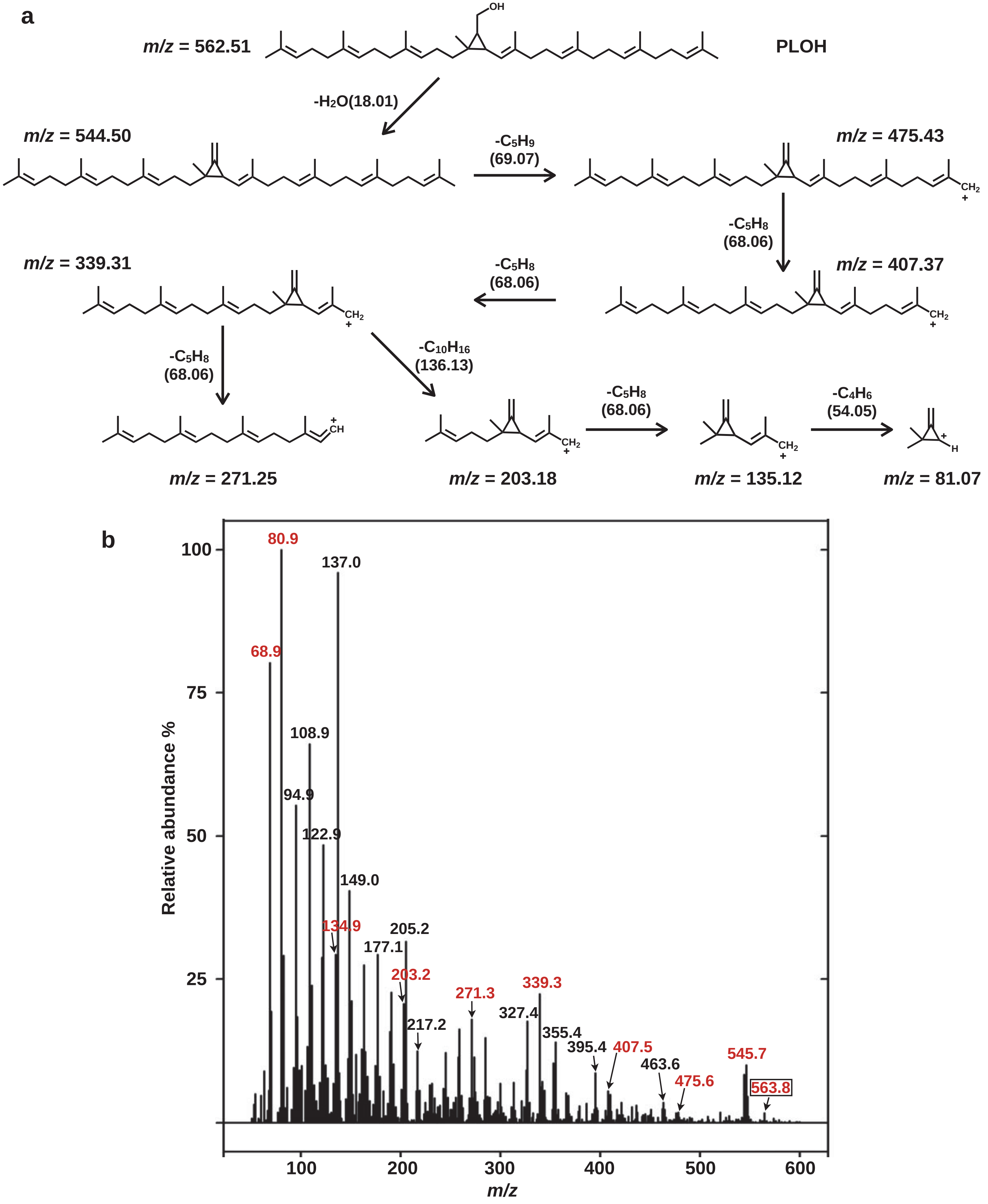

Supplementary Figure 21. **a**, Expected mass spectral fragmentation pattern of prelycopaoctaene alcohol (PLOH). Mass fragmentation pattern of PLOH shown above is adapted from Quershi et al, *J Biol Chem*. **248**:2755-2767 (1973). **b**, The mass spectrum of PLOH molecule when analyzed in chemical ionization mode. Molecular ion in box corresponds to the parent ion of the PLOH molecule and other ionic species in red correspond to fragments of PLOH molecule as shown above in **a**.

δ ^{13}C (δ ^{1}H) chemical shifts

Carbon	Lycopadiene	Lycopatriene	Lycopatetraene	Lycopapentaene	Lycopapentaene Isomer	Lycopahexaene	Lycopaoctaene	$C_{35}H_{64}$
1	22.7 (0.89)	25.7 (1.71)	25.7 (1.71)	25.7 (1.71)	25.7 (1.71)	25.7 (1.71)	25.7 (1.71)	26.0 (1.68)
2	28.0 (1.54)	131.1	131.1	131.1	131.1	131.1	131.1	131.4
3	39.4 (1.16)	125.1 (5.12)	125.2 (5.12)	124.2 (5.15)	124.8 (5.10)	124.3 (5.15)	124.3 (5.15)	124.8 (5.13)
4	24.7 (1.29)	25.6 (1.98)	25.5 (1.99)	26.7 (2.09)	25.5 (1.99)	26.8 (2.06)	27.2 (2.03)	26.3 (2.01)
5	37.4(1.08, 1.28)	37.1 (1.13, 1.34)	37.1 (1.13, 1.34)	39.8 (2.00)	37.1 (1.13, 1.34)	39.8 (1.99)	39.8 (2.00)	40.0 (1.98)
6	32.8 (1.40)	32.7 (1.40)	32.7 (1.40)	135.9	32.7 (1.40)	135.9	135.9	135.1
7	37.4 (1.08, 1.28)	37.4 (1.08, 1.28)	37.4 (1.08, 1.28)	124.2 (5.15)	36.6 (1.08, 1.28)	124.3 (5.15)	124.3 (5.15)	124.8 (5.13)
8	24.5 (1.20, 1.34)	24.5 (1.20, 1.34)	24.5 (1.20, 1.34)	26.7 (2.09)	24.5 (1.20, 1.34)	26.8 (2.06)	27.2 (2.03)	26.3 (2.01)
9	37.4 (1.08, 1.28)	37.4 (1.08, 1.28)	39.3 (1.95)	39.8 (2.00)	39.8 (2.00)	39.8 (1.99)	39.8 (2.00)	40.0 (1.98)
10	31.2 (2.17)	30.8 (2.16)	135.9	135.9	135.9	135.9	135.9	135.1
11	36.8 (1.09, 1.27)	36.8 (1.09, 1.27)	124.1 (5.15)	124.2 (5.15)	124.2 (5.15)	124.3 (5.15)	124.3 (5.15)	124.8 (5.13)
12	25.4 (1.39)	25.4 (1.39)	25.5 (1.99)	26.7 (2.09)	26.7 (2.09)	26.8 (2.06)	27.2 (2.03)	27.2 (2.06)
13	40.0 (1.96)	40.0 (1.96)	40.0 (1.96)	39.8 (2.00)	39.8 (2.00)	39.8 (1.99)	39.8 (2.00)	27.2 (2.06)
14	135.9	135.9	135.9	135.9	135.9	135.9	135.9	124.8 (5.13)
15	124.0 (5.15)	124.1 (5.15)	124.1 (5.15)	124.2 (5.15)	124.2 (5.15)	124.3 (5.15)	124.3 (5.15)	135.1
16	28.3 (2.04)	28.3 (2.04)	28.3 (2.04)	28.3 (2.04)	28.3 (2.04)	28.3 (2.04)	28.3 (2.04)	40.0 (1.98)
17	28.3 (2.04)	28.3 (2.04)	28.3 (2.04)	28.3 (2.04)	28.3 (2.04)	28.3 (2.04)	28.3 (2.04)	24.4 (1.62)
18	124.0 (5.15)	124.1 (5.15)	124.1 (5.15)	124.2 (5.15)	124.2 (5.15)	124.3 (5.15)	124.3 (5.15)	37.7 (1.06, 1.24)
19	135.9	135.9	135.9	135.9	135.9	135.9	135.9	33.0 (1.36)
20	40.0 (1.96)	40.0 (1.96)	40.0 (1.96)	39.8 (2.00)	39.8 (2.00)	39.8 (1.99)	39.8 (2.00)	37.7 (1.06, 1.24)
21	25.4 (1.39)	25.4 (1.39)	25.4 (1.39)	25.4 (1.39)	26.7 (2.09)	26.8 (2.06)	27.2 (2.03)	25.1 (1.21)
22	36.8 (1.09, 1.27)	36.8 (1.09, 1.27)	36.6 (1.08, 1.28)	36.6 (1.08, 1.28)	124.2 (5.15)	124.3 (5.15)	124.3 (5.15)	37.7 (1.06, 1.24)
23	31.2 (2.17)	30.8 (2.16)	30.8 (2.16)	31.0 (2.23)	135.9	135.9	135.9	33.0 (1.36)
24	37.4 (1.08, 1.28)	37.4 (1.08, 1.28)	37.4 (1.08, 1.28)	37.4 (1.08, 1.28)	39.8 (2.00)	39.4 (1.96)	39.8 (2.00)	37.7 (1.06, 1.24)
25	24.5 (1.20, 1.34)	24.5 (1.20, 1.34)	24.5 (1.20, 1.34)	24.5 (1.20, 1.34)	24.5 (1.20, 1.34)	24.5 (1.20, 1.34)	27.2 (2.03)	24.8 (1.19, 1.32)
26	37.4 (1.08, 1.28)	37.4 (1.08, 1.28)	37.4 (1.08, 1.28)	37.4 (1.08, 1.28)	37.4 (1.08, 1.28)	36.6 (1.08, 1.28)	124.3 (5.15)	39.6 (1.13)
27	32.8 (1.40)	32.7 (1.40)	32.7 (1.40)	32.8 (1.40)	32.7 (1.40)	32.8 (1.40)	135.9	28.2 (1.52)
28	37.4 (1.08, 1.28)	37.4 (1.08, 1.28)	37.4 (1.08, 1.28)	37.4 (1.08, 1.28)	37.4 (1.08, 1.28)	37.4 (1.08, 1.28)	39.8 (2.00)	22.9 (0.86)
29	24.7 (1.29)	24.7 (1.29)	24.7 (1.29)	24.7 (1.29)	24.7 (1.29)	24.7 (1.29)	27.2 (2.03)	17.9 (1.61)
30	39.4 (1.16)	39.4 (1.16)	39.4 (1.16)	39.4 (1.16)	39.4 (1.16)	39.4 (1.16)	124.3 (5.15)	16.3 (1.61)
31	28.0 (1.54)	28.0 (1.54)	28.0 (1.54)	28.0 (1.55)	28.0 (1.55)	28.0 (1.55)	135.9	16.3 (1.61)
32	22.7 (0.89)	22.7 (0.89)	22.7 (0.89)	22.7 (0.89)	22.7 (0.89)	22.7 (0.89)	25.7 (1.71)	16.3 (1.61)
33	22.7 (0.89)	17.6 (1.63)	17.6 (1.63)	17.7 (1.63)	17.7 (1.63)	17.7 (1.63)	17.7 (1.63)	20.0 (0.83)
34	19.7 (0.87)	19.7 (0.87)	19.7 (0.87)	16.0 (1.62)	19.8 (0.87)	16.0 (1.61)	16.0 (1.62)	20.0 (0.83)
35	19.7 (0.87)	19.7 (0.87)	15.9 (1.61)	16.0 (1.62)	16.0 (1.62)	16.0 (1.61)	16.0 (1.62)	22.9 (0.86)
36	15.9 (1.61)	15.9 (1.61)	15.9 (1.61)	16.0 (1.62)	16.0 (1.62)	16.0 (1.61)	16.0 (1.62)	
37	15.9 (1.61)	15.9 (1.61)	15.9 (1.61)	16.0 (1.62)	16.0 (1.62)	16.0 (1.61)	16.0 (1.62)	
38	19.7 (0.87)	19.7 (0.87)	19.7 (0.87)	19.8 (0.87)	16.0 (1.62)	16.0 (1.61)	16.0 (1.62)	
39	19.7 (0.87)	19.7 (0.87)	19.7 (0.87)	19.8 (0.87)	19.8 (0.87)	19.8 (0.87)	16.0 (1.62)	
40	22.7 (0.89)	22.7 (0.89)	22.7 (0.89)	22.7 (0.89)	22.7 (0.89)	22.7 (0.89)	17.7 (1.63)	

Supplementary Table 1. ^{13}C and ^{1}H (in parentheses) chemical shifts determined for hydrocarbons from race L. Carbon numbering from 1 to 40 and 1 to 35 is the same as shown for lycopadiene and $C_{35}H_{64}$ in Figure 2A, panel I and VII, respectively.
